# Translation of the Fugl-Meyer assessment into Romanian: Transcultural and semantic-linguistic adaptations and clinical validation

**DOI:** 10.3389/fneur.2022.1022546

**Published:** 2023-01-05

**Authors:** Gelu Onose, Aurelian Anghelescu, Anca Ionescu, Ligia Gabriela Tataranu, Aura Spînu, Ana Maria Bumbea, Corneliu Toader, Sorin Tuţă, Roxana O. Carare, Cristina Popescu, Constantin Munteanu, Collaborative Working Group, Mihaela Oprea, Cristina Daia

**Affiliations:** ^1^Faculty of Medicine, “Carol Davila” University of Medicine and Pharmacy, Bucharest, Romania; ^2^Teaching Emergency Hospital “Bagdasar-Arseni,” Bucharest, Romania; ^3^Faculty of Medicine, University of Medicine and Pharmacy, Craiova, Romania; ^4^Faculty of Medicine, University of Southampton, Southampton, United Kingdom; ^5^Faculty of Medical Bioengineering, University of Medicine and Pharmacy “Grigore T. Popa” Iasi, Iasi, Romania

**Keywords:** Fugl-Meyer assessment scale, upper extremity, lower extremity, translation, semantics, validation study, observer variation

## Abstract

**Purpose:**

The Fugl-Meyer Assessment (FMA) scale, which is widely used and highly recommended, is an appropriate tool for evaluating poststroke sensorimotor and other possible somatic deficits. It is also well-suited for capturing a dynamic rehabilitation process. The aim of this study was to first translate the entire sensorimotor FMA scale into Romanian using the transcultural and semantic-linguistic adaptations of its official afferent protocols and to *then* validate it using the preliminary clinical evaluation of inter- and intra-rater reliability and relevant concurrent validity.

**Methods:**

Through three main steps, we completed a standardized procedure for translating FMA's official afferent evaluation protocols into Romanian and their transcultural and semantic-linguistic adaptation for both the upper and lower extremities. For relevant clinical validation, we evaluated 10 patients after a stroke two times: on days 1 and 2. All patients were evaluated simultaneously by two kinesi-physiotherapists (generically referred to as KFT1 and KFT2) over the course of 2 consecutive days, taking turns in the roles of an examiner and observer, and *vice versa* (inter-rater). Two scores were therefore obtained and compared for the same patient, i.e., being afferent to an inter-rater assay by comparing the assessment outcomes obtained by the two kinesi-physiotherapists, in between, and respectively, to the intra-rater assay: based on the evaluations of the same kinesi-physiotherapist, in two consecutive days, using a rank-based method (Svensson) for statistical analysis. We also compared our final Romanian version of FMA's official protocols for concurrent validity (Spearman's rank correlation statistical method) to both of the widely available assessment instruments: the Barthel Index (BI) and the modified Rankin scale (mRS).

**Results:**

Svensson's method confirmed overall good inter- and intra-rater results for the main parts of the final Romanian version of FMA's evaluation protocols, regarding the percentage of agreement (≥80% on average) and for disagreement: relative position [RP; values outside the interval of (−0.1, 0.1) in only two measurements out of the 56 comparisons we did], relative concentration [RC; values outside the interval of (−0.1, 0.1) in only nine measurements out of the same 56 comparisons done], and relative rank variation [RV; all values within an interval of (0, 0.1) in only five measurements out of the 56 comparisons done]. High correlation values were obtained between the final Romanian version of FMA's evaluation protocols and the BI (ρ = 0.9167; *p* = 0.0002) for FMA–upper extremity (FMA-UE) total A-D (motor function) with ρ = 0.6319 and for FMA-lower extremity (FMA-LE) total E-F (motor function) with *p* = 0.0499, and close to the limit, with the mRS (ρ = −0.5937; *p* = 0.0704) for FMA-UE total A-D (motor function) and (ρ = −0.6615; *p* = 0.0372) for FMA-LE total E-F (motor function).

**Conclusions:**

The final Romanian version of FMA's official evaluation protocols showed good preliminary reliability and validity, which could be thus recommended for use and expected to help improve the standardization of this assessment scale for patients after a stroke in Romania. Furthermore, this endeavor could be added to similar international translation and cross-cultural adaptations, thereby facilitating a more appropriate comparison of the evaluation and outcomes in the management of stroke worldwide.

## Background and purpose

Stroke is classically defined as “rapidly developed clinical signs of focal (or global) disturbance of cerebral function, lasting more than 24 h or resulting in death, with no apparent cause other than vascular origin” (1), which includes cerebral infarction, intracerebral hemorrhage, and subarachnoid hemorrhage (2). Stroke is a frequently occurring condition that increases especially with age (3) and often becomes severe, even life-threatening, with marked disabling potential (4) and, therefore, with great impact on the individuals and their families, as well as in society. It is estimated that such conditions cause “nearly” 800,000 cases (new and recurrent) per year in the USA (5) and more than 610,000 new cases/year in Europe (6), accounting them the second most common cause of death worldwide (7), and—globally—the third cause of (lost) disability-adjusted life years (DALYs) (8). More than a quarter of stroke survivors (26%) develop permanent neurological deficits that negatively affect their autonomy in daily living activities and their overall mobility “due to hemiparesis” (7). Mobility impairments are considered to be key aspects: therefore, also among the top 10 research priorities related to life after stroke, for patients and their caregivers, as well as for clinicians and health professionals (9). Rehabilitation is considered an essential intervention in stroke care and—especially as “community-based rehabilitation”—in reducing stroke-related costs (10). Although poststroke natural motor recovery has been classically considered to follow a well-grounded and stepwise sequence from flaccidity to spasticity and ultimately to motor recovery (11, 12), there is a need to approach a “heterogeneous group” of stroke conditions (13), based on precise/standardized, and dedicated clinometric instruments (12). Despite a consensus among published guidelines recommending the use of valid and reliable assessment tools, “it is not clear which outcome measure (OM) should be selected for a particular need” (14). Therefore, it is important to support the implementation of an overall, clinically efficient management as we continue to explore and use appropriate and related measurement instruments in this domain. Clinicians and researchers must have access to “reliable measures of the concepts of interest in their own cultures and languages to provide high-quality patient care” (15). Such an appropriate tool to evaluate post-stroke sensorimotor and other possible somatic deficits is the widely used and highly recommended Fugl-Meyer Assessment (FMA) scale (12, 16, 17). The FMA scale measures post-stroke impairments and is therefore well-suited for capturing a dynamic process of rehabilitation. The motor domain of the FMA scale has consistent validity, very good intra- and inter-rater reliability, and may be used in both clinical trials and community hospital settings (12).

This study aimed to translate the entire (sensorimotor) FMA scale into Romanian with relevant transcultural and semantic-linguistic adaptations and clinical validation. This process has so far been completed in Italian, Spanish, Greek, Ukrainian, Swedish, Norwegian, Danish, Latvian, Urdu, and Korean ^1^. For our endeavor of translation, transcultural, and semantic-linguistic adaptations from English to Romanian of FMA's official afferent evaluation protocols, we have been granted approval and support from the official administrators of this scale, at the University of Gothenburg ^1^. We therefore herein present the first Romanian version of the entire FMA scale (i.e., the official afferent evaluation protocols), for the upper extremity (UE), and also for the lower extremity (LE), which we clinically validated in a group of patients with subacute, subchronic, or chronic post-stroke hemiparesis, without significant somatosensory, cognitive, or speech impairments.

## Materials and methods

**Figure 1:**
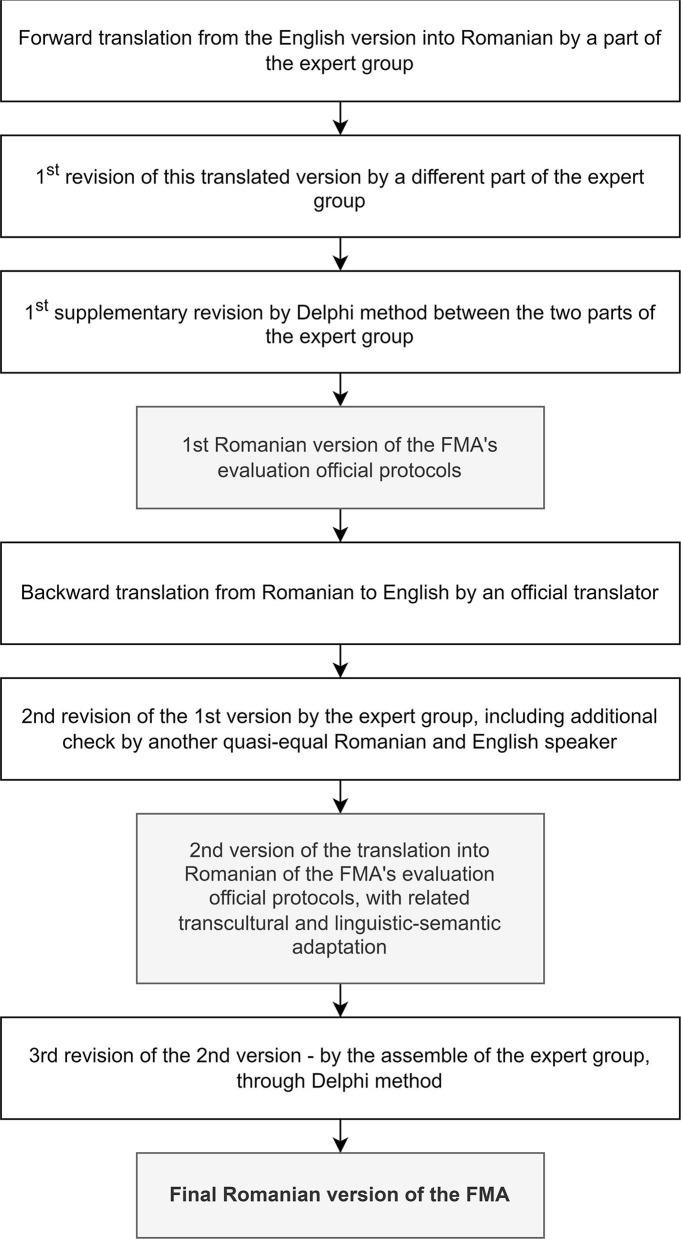
The flowchart of our endeavor: a step-by-step translation of FMA's official evaluation protocols into Romanian, with relevant transcultural and semantic-linguistic adaptation.

In the first part of the work (see Annex 1: the official evaluation protocols in English provided by their official administrator: Gothenburg University, and Annex 2: the final translated version of FMA's official evaluation protocols into Romanian, with relevant transcultural and semantic-linguistic adaptations), we fulfilled the abovementioned process for both UE and LE and went through the following steps [including in the adaptive consideration of the World Health Organization—Guidelines on Translation—The process of translation and adaptation of instruments (18)] (Figure 1):

1. Forward translation of FMA's official evaluation protocols from English to Romanian by a member of our expert group. The first revision of this forward translated version was independently carried out by a different member of our expert group (a native Romanian speaker who is clinically qualified, academically fluent in English, and has worked at an international level in the UK for the last 25 years). The first addition/supplementary revision using the Delphi method was achieved from the two expert group members mentioned earlier;
2. backward translation of the first Romanian version (version 1) of FMA's official afferent evaluation protocols—from Romanian to English—by an independent official translator (i.e., from a prestigious company specialized in translations);
3. the second revision of the first Romanian version of the FMA scale with relevant linguistic-semantic checks and adaptations: through cross-analysis by an expert group, including additional checks by another quasi-equivalent Romanian and English speaker (a Romanian physician who has lived and worked in the UK for the last 15 years)—the second Romanian version (version 2) of the translation of FMA's official evaluation protocols; and
4. the third revision—that is, the second version of the translation of FMA's official afferent evaluation protocols into Romanian by an ensemble of the expert group through the Delphi method—“forward and backward translation, stepwise reviewing by bilingual and professional experts to ensure conceptual and semantic equivalence” (19)—the final (operational) translated version of FMA's official evaluation protocols into Romanian, with relevant transcultural and semantic-linguistic adaptations [“The cross-cultural adaptation process is important when an instrument is used in a different language, setting, and time to reduce the risk of introducing bias into a study” (20)], noted shortly: *Final Romanian version of the FMA*.

Further, this was used in a validation pilot trial in patients after a stroke (in accordance with and including the counseling of the official administrators of the FMA scale at Gothenburg University).

## Afferent validation pilot study

### Study design

We enrolled 10 patients with post-stroke—who were admitted to the Neuromuscular Clinic Division of the Teaching Emergency Hospital “Bagdasar-Arseni,” in Bucharest, Romania and to the National Institute of Neurology and Neuro-Vascular Diseases, between April 2021 and July 2021.

We fulfilled the preliminary procedures related to Bio-Ethics, including an individual written informed consent, signed by each patient. We evaluated the abovementioned 10 patients after a stroke at two time points: days 1 and 2.

#### Inclusion criteria

Patients with subacute, subchronic (minimum 3 weeks, maximum 6 weeks from acute cerebrovascular accident—CVA/stroke/brain attack) hemiparetic post-stroke, with patients age being ≥18 years.

#### Exclusion criteria

Patients with poor general health (including neurological conditions), severe sensory impairments (tactile, proprioceptive, balance, coordination, visual, and auditory), marked communication (aphasia, especially including receptive elements), and/or cognitive problems [Montreal Cognitive Assessment (MoCA) < 17 points, see below], the complete or segmentary absence of (a) limbs/(s), and any other aspect that could negatively affect the patient's engagement in this type of assessment.

The characteristics of the sample included in the clinical validation study are presented in Table 1.

**Table 1:** The characteristics of the sample included in the clinical validation study—ID, identification.

**Crt. No. ID**	**Age (years)**	**Gender**	**Stroke type and location**	**Lesion side**	**mRS (0–6)**	**BI (0–100)**	**MoCA (0–30)**	FMA UE (0–66)^*^	FMA LE (0–34)^*^	**Date of acute stroke**
1	80	M	Ischaemic basal ganglia	R	3	40	20	47	28	22/04/2021
2	46	M	Haemorrhagic, thalamus, mesencephalon	L	3	40	21	44	26	14/04/2021
3	78	M	Ischaemic ACA	R	4	30	18	29	13	22/06/2021
4	72	M	Ischaemic basal ganglia	L	4	35	18	22	27	17/05/2021
5	58	F	Ischaemic MCA	L	3	48	21	44	26	02/05/2021
6	69	F	Ischaemic ACA	L	3	49	20	62	30	04/06/2021
7	63	M	Hemorrhagic, frontal	L	4	40	19	63	12	10/07/2021
8	66	F	Ischaemic MCA	R	4	20	17	0	17	01/05/2021
9	39	M	Ischaemic paramedian pontine	R	3	39	18	40	26	12/04/2021
10	67	M	Ischaemic MCA	R	4	38	19	12	23	03/07/2021

ID, identification number; F, female; M, male; mRS, modified ranking scale; BI, Barthel index; MoCA, Montreal cognitive assessment; FMA-UE, Fugl-Meyer assessment-upper extremity; FMA-LE, Fugl-Meyer assessment-lower extremity; MCA, middle cerebral artery; PCA, posterior cerebral artery; ACA, anterior cerebral artery.

* The data present the scores obtained during the first assessment (day 1).

We validated the final Romanian version of the FMA protocols, as well as any possible disturbances in their practical and clinical use due to the translation and relevant necessary transcultural and semantic-linguistic adaptations. Thus, on the one hand, we applied the Svensson statistical^2^ rank-based “approach of observations” method (21) that “focuses on the differences in the ranking approaches between the measures of association and of disagreement in paired ordinal data,” (22) assessing the inter-rater and, respectively, intra-rater reliability of our primary data, and, on the other hand, we compared our final version of FMA with widely used assessment tools, such as, the Barthel index (BI) (23) and the modified Rankin scale (mRS) ^3^, using Spearman's rank correlation statistical method (24).

#### Inter- and intra-rater assays

Each patient has been evaluated by two knowledgeable licensed kinesi(physio)therapists trained for the FMA scale: simultaneously (i.e., directly by one and indirectly by the other) but independently, for two consecutive days, under the guidance of a Physical and Rehabilitation Medicine (PRM) physician within our multiprofessional staff. This was conceptually consistent with the requirement that “standardized measurement methods and training of therapist assessors for a multi-site, rehabilitation, randomized, clinical trial resulted in high inter-rater reliability for the Fugl-Meyer motor and sensory assessments” (25). Kinesi(physio)therapists are referred to as “KT 1” and “KT 2.”

One of them effectively examined and scored the patient through the final Romanian version of FMA's evaluation protocols (examiner KT 1), while the other (examiner KT 2) observed this evaluation (performed by the examiner KT 1) and, based on the respective observation (noted: day 1), scored the FMA scale for the same patient, without communication between the two examiners, neither at the moment of the assessment nor afterward. In fact, the results of FMA's evaluation protocols obtained for each patient assessed will remain unknown to each of the two assessors. On the next day, another session of the evaluation (noted as day 2), the same examiners (KT 1 and KT 2) proceeded in the same way but reversed their roles. Consequently, in the evaluation on day 2, KT 1 was an observer and KT 2 was an examiner.

Each team consisting of two examiners (generically referred to as KT 1 and KT 2) evaluated the patients according to the final Romanian version of FMA's protocols, rating in accordance with the specific values/points afferent to its parts/steps, as follows:

For the UE, we evaluated (see Annex 1 and respectively, Annex 2—with relevant specifications added regarding the latter):

- A = Upper Extremity, sitting position/“Extremitatea Superioară, din poziţie şezând”
- [-] B = Wrist/“încheietura mâinii”
- C = Hand/“Mâna”
- D = Coordination/Speed/“(Dis)Coordonare/Viteză”
- Total points A–D/“Total puncte A–D”
- H = Sensation upper extremity/“Sensibilitate extremitatea superioară”
- I = Passive joint motion/“Mobilitate articulară pasivă”
- J = Joint pain/“Durere articulară”

Total points FMA-UE/“Total puncte scala Fugl-Mayer Extremitatea Superioară.”

For the LE we noted (see Annex 1 and respectively, Annex 2—with related specifications added regarding the latter):

- E = Lower Extremity/“Extremitatea Inferioară”
- F = Coordination/Speed/“Coordonare/Viteză”
- Total points E–F/“Total puncte E–F”
- H = Sensation lower extremity/“Sensibilitate extremitatea inferioară”
- I = Passive joint motion/“Mobilitate articulară pasivă”
- J = Joint pain/“Durere articulară”
- Total points FMA-LE/“Total puncte scala Fugl-Mayer Extremitatea Inferioară.”

Thus, for the same patient, two scores were obtained. The inter-rater assay was obtained by comparing the assessment's results of KT 1 and KT 2 on days 1 and 2. The intra-rater assay resulted in the comparison of the evaluation outcomes of the same kinesi(physio)therapist, in 2 consecutive days (days 1 and 2).

To compare the concurrent validity of our final Romanian version of FMA's evaluation protocols with clinically and functionally quantified assessment instruments, we used

- the BI (23), as a reference —“gold standard;”
- the mRS ^3^, as a source of the overall disability status of each recruited patient.

For evaluating the cognitive state of our enrolled patients to underpin the exclusion criteria, we availed the MoCA ^4^.

The assay of all enrolled patients for stratification/verification for the exclusion criteria, the last mentioned three scales (BI, mRS, and MoCA) were performed by a PRM physician within our staff.

### Statistical analysis afferent to the validation processing endeavors/procedures

Svensson's method ^2^ was applied for objective assessment and quantification of the inter- and intra-rater reliability and was recommended to determine the consensus level or percentage of agreement (PA) between the “two different raters (during the same session)” and between the first and second observation (for each rater) (26). We considered a 70–80% agreement satisfactory (27). The systematic disagreement between the evaluators is referred to as “the RP (o.n.), the RC (o.n.), and the RV (o.n.);” more specifically, for the first two abovementioned statistical parameters, values “from −1 to 1, where 0 means no difference between evaluators,” and “within −0.1 and 0.1” were deemed as inessential clinical relevance (19), whereas the values outside the range −0.1 and 0.1 may be considered as disagreements that are clinically relevant and concerning the last statistical parameter; this may vary from 0 to 1 [“ <0.1 means that the difference is negligible” (19)—all with the consequent statistical significance considered where “the 95% confidence interval (CI) that did not include the value 0” (26)].

We used Spearman's rank correlation method for the afferent statistical approach to compare the concurrent validity of our final Romanian version of FMA's evaluation protocols with the widely recognized abovementioned evaluation instruments (24).

## Results

### Inter- and intra-rater assessment through the Svensson statistical method

In Table 2, we synthetically present the related assay outcomes.

**Table 2:** Inter- and intra-rater assessment outcomes obtained through Svensson's statistical method.

**Comparison Type**	**Extremity**	**FMA scale part**	**Approach**	**PA**	**RP**	**RC**	**RV**	**Svensson's template used**
**INTER-rater (KT1 vs KT2)**	**Upper extremity**	**A**	Day1	60%	0.08	0.016536	0.012	VAS scatterplot
			Day2	70%	−0.01	0.016162	0.012	
		**B**	Day1	80%	0.04	−0.13369	0	11 categories
			Day2	80%	0.04	0.035165	0	
		**C**	Day1	90%	0.05	0.065934	0.036	VAS scatterplot
			Day2	100%	0	0	0	
		**D**	Day1	90%	−0.03	0.09009	0	11 categories
			Day2	100%	0	0	0	
		**Total A-D (motor function)**	Day1	40%	0.04	0.004026	0.024	VAS scatterplot
			Day2	50%	0.03	0.008081	0	
		**H**	Day1	100%	0		0	VAS scatterplot
			Day2	100%	0		0	
		**I**	Day1	100%	0	0	0	VAS scatterplot
			Day2	90%	−0.01	−0.06378	0	
		**J**	Day1	100%	0	0	0	VAS scatterplot
			Day2	90%	−0.01	−0.03838	0	
	**Lower extremity**	**E**	Day1	60%	0	0.064646	0.06	VAS scatterplot
			Day2	70%	−0.01	0.012121	0.036	
		**F**	Day1	90%	0.07	−0.03166	0	11 categories
			Day2	100%	0	0	0	
		**Total E-F (motor function)**	Day1	60%	−0.01	0.048701	0.168	VAS scatterplot
			Day2	70%	0	−0.06039	0.012	
		**H**	Day1	100%	0	0	0	VAS scatterplot
			Day2	100%	0	0	0	
		**I**	Day1	100%	0	0	0	VAS scatterplot
			Day2	100%	0	0	0	
		**J**	Day1	90%	0.03	0.178571	0.012	VAS scatterplot
			Day2	100%	0	0	0	
**INTRA-rater (Day1 vs Day2)**	**Upper extremity**	**A**	KT1	50%	0.14	−0.07083	0.192	VAS scatterplot
			KT2	40%	0.04	−0.23133	0.012	
		**B**	KT1	80%	−0.04	−0.03516	0	11 categories
			KT2	80%	−0.04	0.13369	0	
		**C**	KT1	80%	0	0.168138	0.036	VAS scatterplot
			KT2	90%	−0.05	0.108507	0	
		**D**	KT1	70%	0.09	0.101099	0.048	11 categories
			KT2	80%	0.12	0.008913	0.036	
		**Total A-D (motor function)**	KT1	30%	−0.02	−0.00803	0.108	VAS scatterplot
			KT2	30%	0.05	−0.07246	0.048	
		**H**	KT1	100%	0		0	VAS scatterplot
			KT2	100%	0		0	
		**I**	KT1	90%	0.01	0.063785	0	VAS scatterplot
			KT2	100%	0	0	0	
		**J**	KT1	80%	0.08	−0.07675	0	VAS scatterplot
			KT2	90%	0.07	−0.11513	0	
	**Lower extremity**	**E**	KT1	50%	0.1	−0.08681	0.204	VAS scatterplot
			KT2	50%	0.1	−0.18333	0.096	
		**F**	KT1	80%	0.08	−0.07237	0	11 categories
			KT2	90%	0.01	−0.03906	0	
		**Total E-F (motor function)**	KT1	50%	0.09	−0.11084	0.084	VAS scatterplot
			KT2	50%	0.1	−0.2439	0.144	
		**H**	KT1	100%	0	0	0	VAS scatterplot
			KT2	100%	0	0	0	
		**I**	KT1	90%	0.02	0.119048	0	VAS scatterplot
			KT2	90%	0.02	0.119048	0	
		**J**	KT1	90%	0.03	0.178571	0.012	VAS scatterplot
			KT2	100%	0	0	0	

Synthesis Table. Svensson's method results: inter- and intra-rater comparisons.

### Concurrent validity with other clinical and functional evaluation instruments

To perform the correlation test afferent to objectifying concurrent validity FMA score values, these were computed by averaging the score values four times (two times for each KT—see Materials and Methods) regarding the total UE A-D (motor function) and, similarly, the total LE E-F (motor function). The correlation values obtained were very good for BI and close to the limit for the mRS (Table 3).

**Table 3:** Spearman correlation test results.

	**FMA UE A-D**		**FMA LE E-F**	
	**ρ**	***p*-value**	**ρ**	***p*-value**
BI	0.916962	0.000188	0.631949	0.04998
mRS	−0.59367	0.07039	−0.6615	0.03724

## Discussion

In this study, we followed a rigorous, standardized procedure of translation, including transcultural and semantic-linguistic adaptations, of the original FMA official evaluation protocols from English into Romanian. The final translated version of the FMA scale in Romanian was a gradual process based on the relevant experience of other countries and the following three classical steps: first, forward translation with two revisions; second, backward with transcultural and semantic-linguistic adaptations; and third, integrative-conceptual revision (by a consensus method), thus providing the verified final Romanian version of the FMA scale.

Details/specifications regarding the translation process were:

- Afferent to the FMA-UE:

1. (“A. Upper Extremity, sitting position... II. Volitional movement.... Flexor synergy.... external rotation”): “abducţie şi rotatie externă–articulaţia scapulo-humerală.”
2. (“A. Upper Extremity, sitting position... II. Volitional movement.... Extensor synergy.... Shoulder adduction/internal rotation”): “Umăr/adducţie/rotaţie interna—poate fi susţinut braţul pentru a lua/menţine poziţia de start.
  - “prezenţa doar a mişcărilor compensatorii în locul celor active se cuantifică/punctează cu 0 (exemple: 3. flexie antebrat şi pronatie, la flexia braţului/umăr 90–180°; 4. abductie si flexie cot la supinaţie).”
3. (“A. Upper Extremity, sitting position... IV. Volitional movement with little or no synergy.... no shoulder abduction or elbow flection”): “abducţie şi flexie cot la supinaţie—i.e., the presence of just compensatory movements instead of active ones is quantified/scored with 0 (examples: 3 forearm flexion and pronation at arm/shoulder 90–180° flexion; 4. Elbow abduction and flection at supination (abducţie şi flexie cot la supinaţie).”
4. (“A. Upper Extremity, sitting position... IV. Volitional movement with little or no synergy.... no pronation/supination, starting position impossible”): “abducţie şi flexie cot la supinaţie”.
5. (“Upper extremity V. Normal reflex activity ... Biceps, triceps, finger flexors”): “hand in pronation patients'fingers in semiflection MCF and IF, relaxed laid on the index and medius of the examiner's finger, when this one percusses overhand with the reflexes hammer his own fingers, it may be obtained a patient's fingers flexion, possibly a normal aspect,” i.e.: “mâna în pronaţie, degetele pacientului in semiflexie MCF şi IF, sprijinite relaxat pe indexul şi mediusul examinatorului, când acesta îşi percută de jos în sus cu ciocanul de reflexe propriile degete, se poate obţine o flexie a degetelor pacientului, aspect posibil normal.”

- Afferent to the FMA-LE:

1. (“L.E. II. ... Flexor synergy: .... Hip Flexion”): abduction/external rotation reckoned to be partial – the presence just of the respective compensatory movements instead of the active ones is quantified/ scored with 0, i.e., “abducţie/rotaţie externă considerate a fi parţiale – prezenţa doar a respectivelor mişcări compensatorii în locul celor active se cuantifică cu 0”
2. (“LE. III. ... Volitional movement mixing synergy ...Knee flexion from actively or passively extended knee”): it is applied light resistence disal posterior at the knee level to make sure of active motion, i.e., se aplică uşoară rezistenţă distal posterior, la nivelul călcâiului, pentru a ne asigura de mişcare activă.”

The pilot evaluation study of the reliability and concurrent validity of the Romanian FMA comprised 10 patients with stroke. As previously described, each of them had seven evaluations: four from two kinesi(physio)therapists (on days 1 and 2) and the remaining three from a PRM physician. We thereby contributed to the “usefulness of this method for clinical assessment and as a tool for the comparative analysis of the effectiveness of various therapeutic interventions” (28). Svensson's method overall confirmed the final Romanian version of FMA's reliability.

In addition to the output of the final Romanian version of FMA's evaluation protocols, the assessments of the enrolled patients emphasized very good statistical results for the BI and borderline for the mRS, mainly due to the small number of items of this latter scale, suggesting that the evaluators applied all the related scales well, with the outcomes being the clinical and functional status of each patient.

A few unexpected outcomes were obtained for total A–D (motor function) and total E–F (motor function) for UE and LE, respectively. They can be explained by the fact that, even though individual percentage agreement results were high, when summed up in some cases (and particularly penalized by the small number of pairs of data), such gaps increased.

The least PA results were obtained for the following scales:

° A, UE, inter-rater;
° Total A–D, UE, inter-rater;
° Total A–D, UE, intra-rater;
° E, LE, inter-rater; and
° Total E–F, LE, inter-rater.

However, disagreement measures were met in most cases, of 56 such comparisons, with 40 in the compliance interval.

## Limitations of this study

Due to the objective situation caused by the COVID-19 pandemic, the sample size was small, 10 post-stroke patients, but this was considered satisfactory for this kind of pilot study, aimed at validating the translation of the FMA scale into Romanian. Therefore, all statistical analyses and data processing were further adapted to the sample size. To perform the correlation test being afferent to objectifying the concurrent validity FMA score values, these were computed by averaging the score values obtained four times (two times for each KFT, see Materials and Methods) with respect to the total UE A–D (motor function), and similarly, the total LE E–F (motor function). Of course, during the COVID-19 pandemic, associated epidemiological cautions and consequent restrictions were eliminated, so we intend to increase the sample size to further validate our translation of the FMA scale.

## Conclusions

Our stated aim was to contribute to the growing extension of the global availability of useful FMA scales by disseminating appropriate translations into different languages, including Romanian. The final Romanian version of the FMA scale (Annex 2) was developed using a standardized translation methodology, which included transcultural and semantic-linguistic adaptations. The preliminary evaluation of reliability and validity was demonstrated in a sample of patients after a stroke. This work contributes to the standardization and wider use of the FMA scale in Romania. Additionally, this achievement, along with the translation of this scale into more languages, tends to enhance efficient professional communication internationally regarding the diagnosis, treatment, and rehabilitation approaches of stroke.

## Data availability statement

The original contributions presented in the study are included in the article/Supplementary material, further inquiries can be directed to the corresponding authors.

## Ethics statement

This article was approved by the Ethics Commission/Council of the Teaching Emergency Hospital Bagdasar-Arseni (No. 4182/10.2.2021) and of the National Institute of Neurology and Neurovascular Diseases (No. 3171/08.05.2021). The patients/participants provided their written informed consent to participate in this study.

## Collaborative working group

Mihaela Oprea^1,2^, Elena Constantin^2^, Mihai Băilă^1,2^, Alexandra Cocoloş^2^, Ioana Elisei^2^, Andrei Stănescu^2^, Andreea Frunză^2^, Aurelia Bichir^2^, Alexandru Pandrea^2^, Florin Marinescu^2^, Cristian Răducanu^2^, Valentina Matei^2^, Ionuţ Colibăşeanu^2^, Ştefan Petre^2^, Diana Church^3^, and Roxana Popa^4^

^1^ Faculty of Medicine, “Carol Davila” University of Medicine and Pharmacy, Bucharest, Romania

^2^ Teaching Emergency Hospital “Bagdasar-Arseni,” Bucharest, Romania

^3^ Living Well Partnership, Southampton, United Kingdom

^4^ GFT IT Consulting, S.L.U., Sant Cugat del Vallès, Spain.

## Author contributions

Conceptualization: GO, AA, CD, and LT. Methodology: GO, CD, RO, AI, AA, and AB. Software: CM and AI. Validation: GO, CD, CP, AS, RO, CT, ST, AI, AB, and CWG. Formal analysis: CD, GO, AA, AB, CT, ST, AI, CP, LT, and CWG. Data curation: CD. Writing—original draft preparation: GO, CD, AB, and AA. Writing—review and editing: GO, CD, AA, CM, AB, AI, CP, LT, and CWG. Visualization: GO, CD, AA, AB, AS, CP, CT, CM, CT, ST, AI, LT, and CWG. Supervision: GO, CD, CM, AB, CP, AA, AS, AI, and CWG. All authors have read and agreed to the published version of the manuscript.

## Footnotes

^1^ https://www.gu.se/en/neuroscience-physiology/fugl-meyer-assessment

^2^ https://avdic.se/svenssonsmethodenglish

^3^ https://epos.myesr.org/posterimage/esr/ecr2019/147892/mediagallery/800774?deliveroriginal=1

^4^ https://www.parkinsons.va.gov/resources/MOCA-Test-English.pdf
