# Supplementary material for: Translation of the Fugl-Meyer assessment into Romanian: Transcultural and semantic-linguistic adaptations and clinical validation
Source: Front Neurol. 2023 Jan 5;13:1022546. doi: 10.3389/fneur.2022.1022546 (PMC9879050; doi:10.3389/fneur.2022.1022546)
Supplement: Supplementary file 1 [file Data_Sheet_1.ZIP › Suppl.2 - Svenssons method detailed results.docx]

## INTER-rater Svensson’s results

The inter-rater Svensson’s method was done comparing values from KT1 with values from KT2 for each of the two days within the evaluation, therefore the method was applied two times (each with the 10 patients), for each of the FMA scale parts. The aim here is to prove that the interpretation of the scale is objective enough, that is, it is independent of whether it is evaluated by one KT or the other. The templates used were 11 categories template for B, D and F scales, and VAS scatterplot for the rest of them.

### A scale Upper Extremity

Given that A scale maximum value is more than 11, we need to use VAS scatterplot template. Below the results:


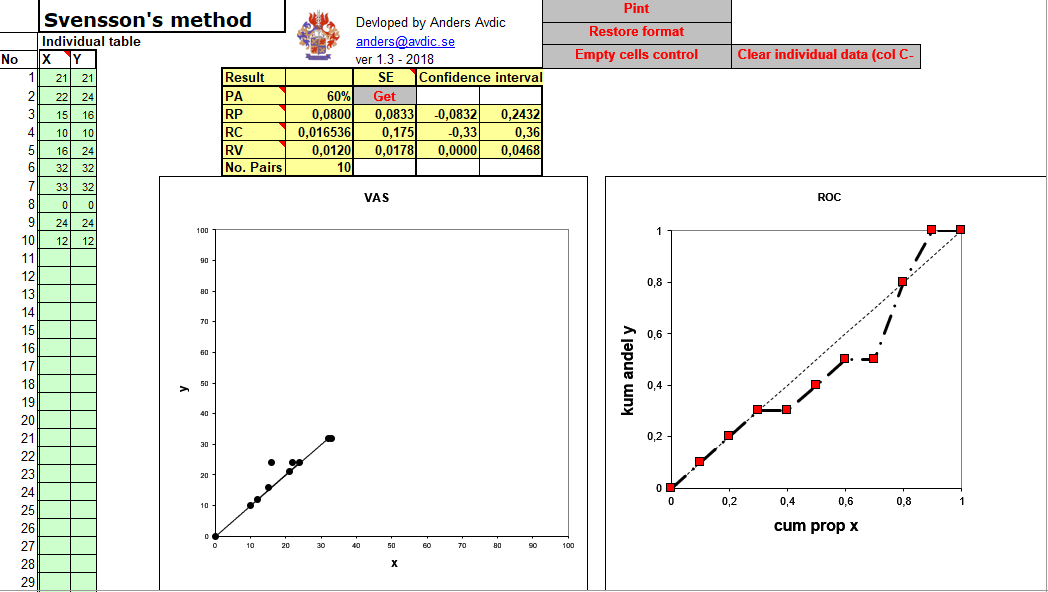
Figure 1. A scale, Upper Extremity inter-rater, day1_scatterplot and ROC curve


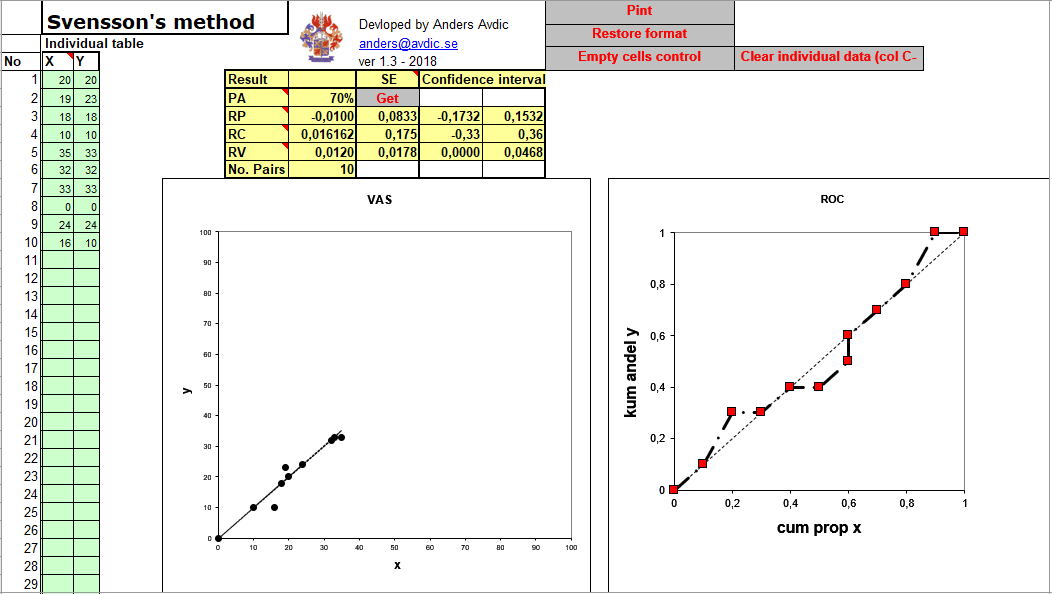
Figure 2. A scale, Upper Extremity inter-rater, day2_ scatterplot and ROC curve

### B scale Upper Extremity

This scale allows 11 possible values/levels, i.e. from 0 to 10, hence the 11-category Svensson template was used. We obtained both times PA of 80%, which is satisfactory. RC only was slightly under -0.1. Below the results:


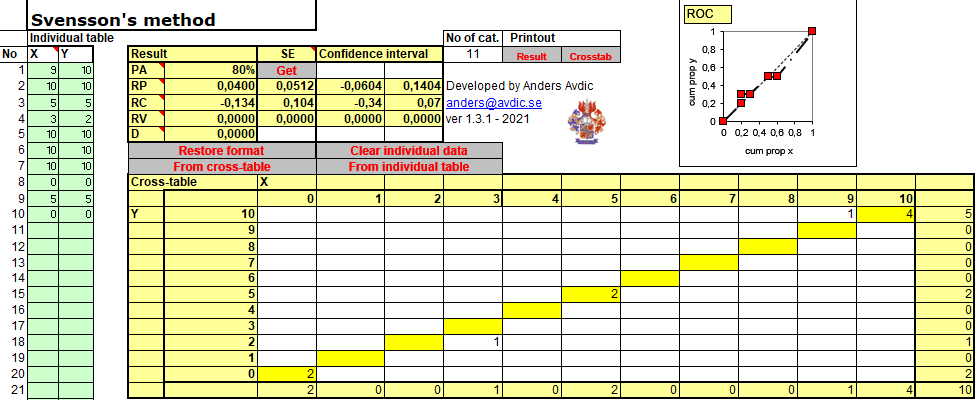


Figure 3. B scale, Upper Extremity inter-rater, day1_crosstab


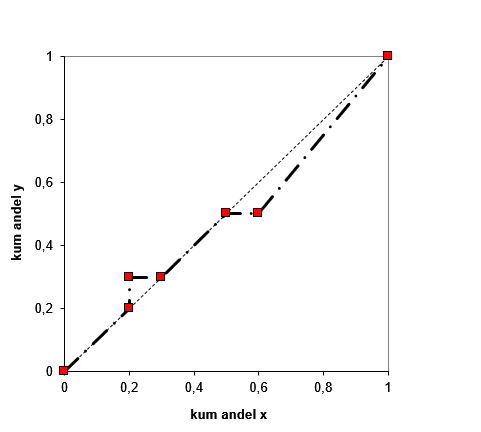


Figure 4. B scale, Upper Extremity inter-rater, day1_ROC curve


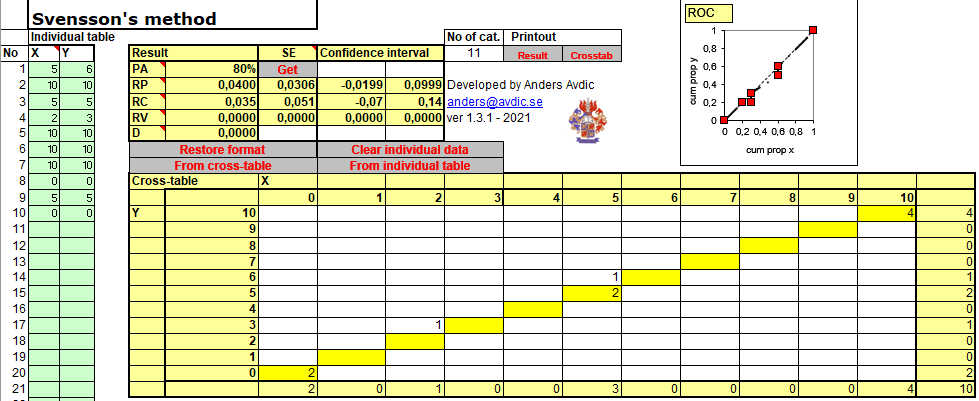


Figure 5. B scale, Upper Extremity inter-rater, day2_crosstab


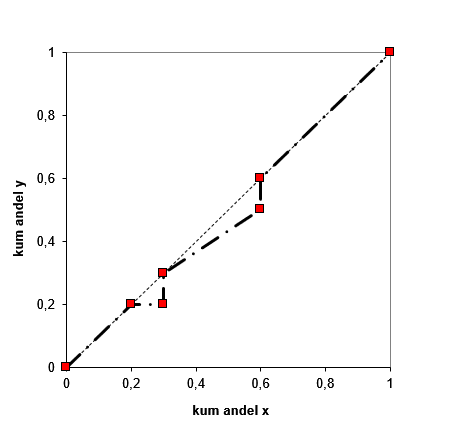


Figure 6. B scale, Upper Extremity inter-rater, day2_ROC curve

### C scale Upper Extremity


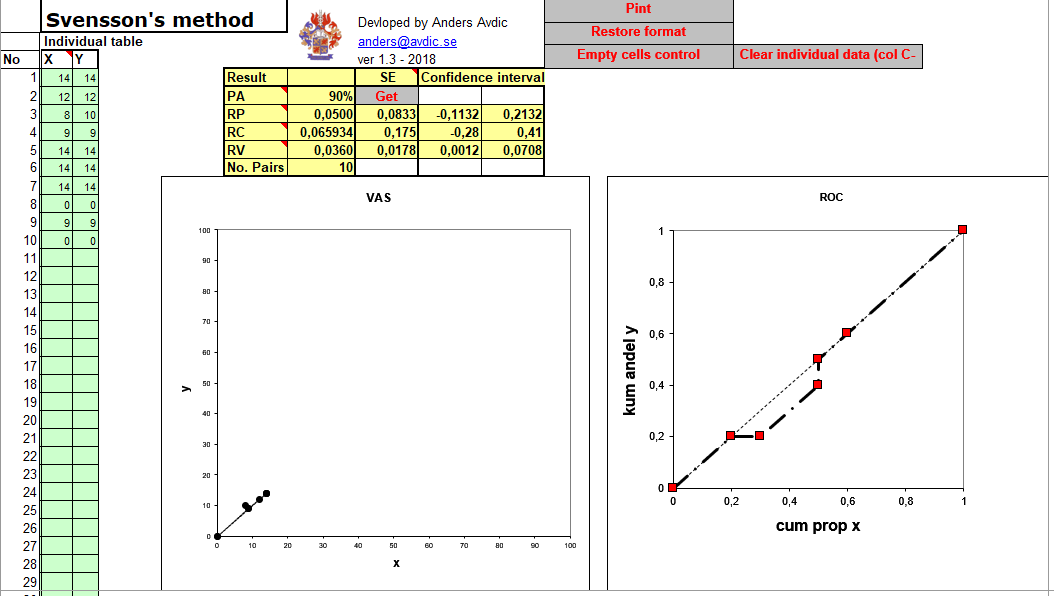
Figure 7. C scale, Upper Extremity inter-rater, day1_ scatterplot and ROC curve


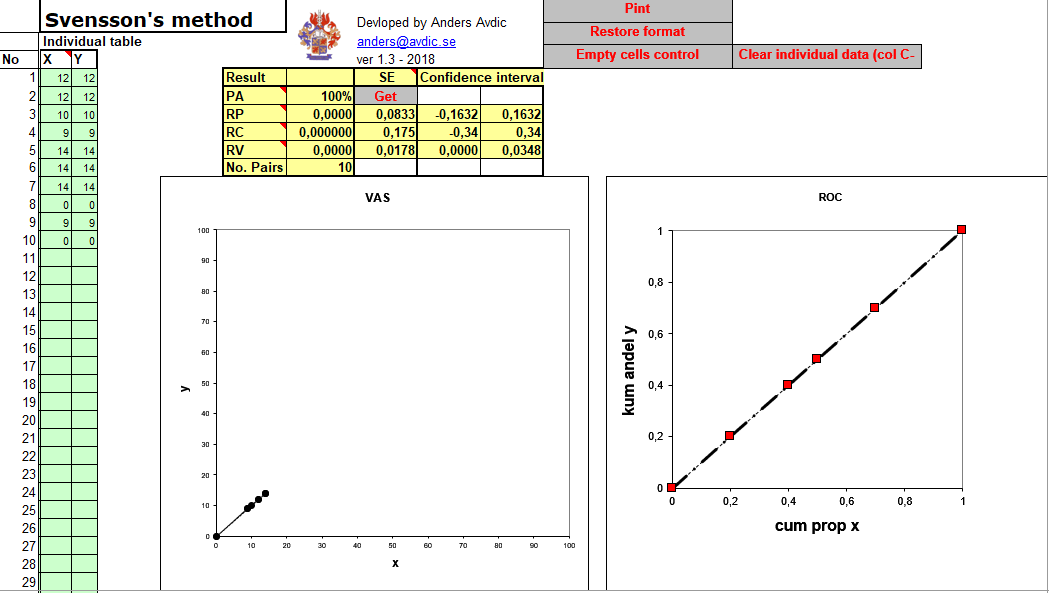
Figure 8. C scale, Upper Extremity inter-rater, day2_ scatterplot and ROC curve

### D scale Upper Extremity

This scale allows 7 possible values, i.e. from 0 to 6, hence the 11-category Svensson template was used. Below the results, the lowest percentage agreement being of 90% (in one case out of the total of 10 patients). Relative Concentration was very good, as well as Relative Position, meeting the interval between -0.1 and 0.1:


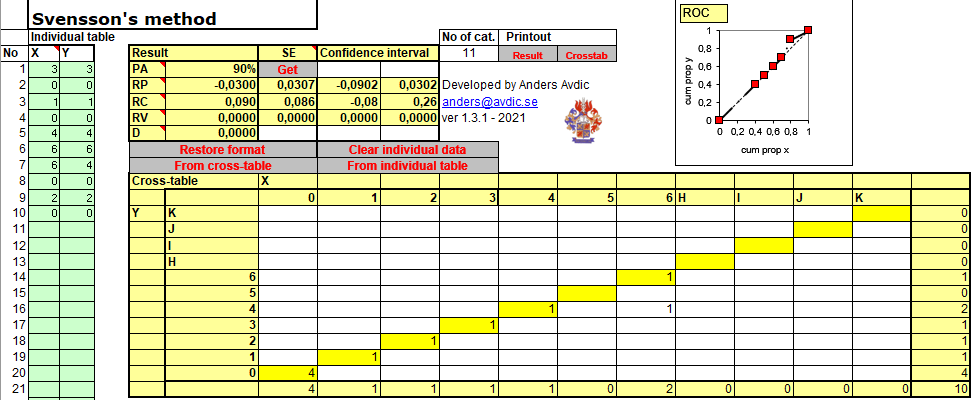


Figure 9. D scale, Upper Extremity inter-rater, day1_crosstab
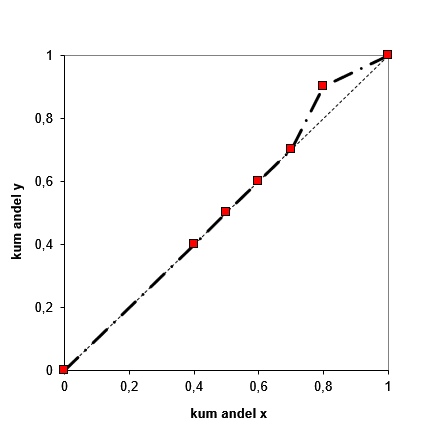


Figure 10. D scale, Upper Extremity inter-rater, day1_ROC curve


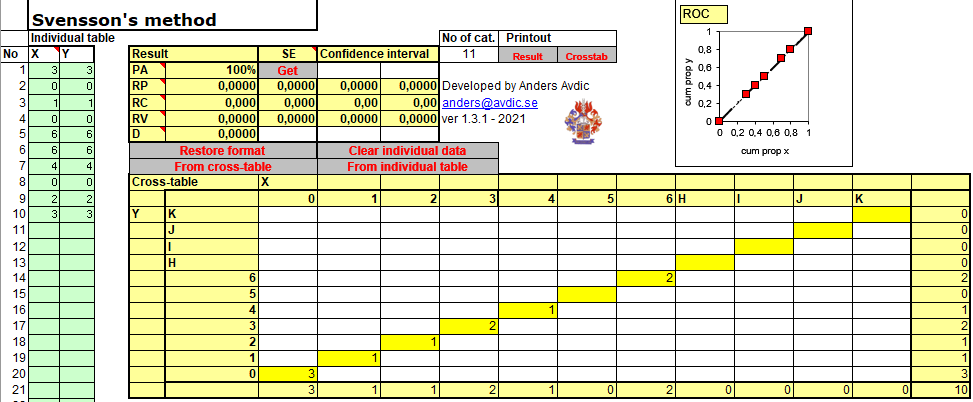
Figure 11. D scale, Upper Extremity inter-rater, day2_crosstab


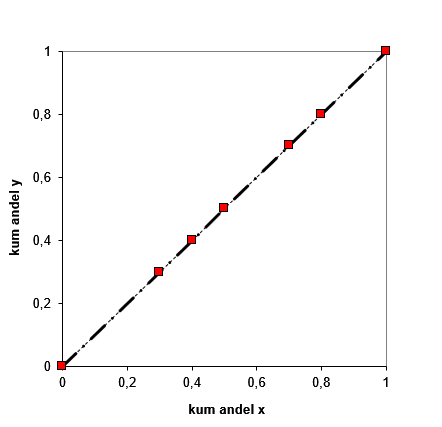


Figure 12. D scale, Upper Extremity inter-rater, day2_ROC curve

### Total A-D Upper Extremity (motor function)


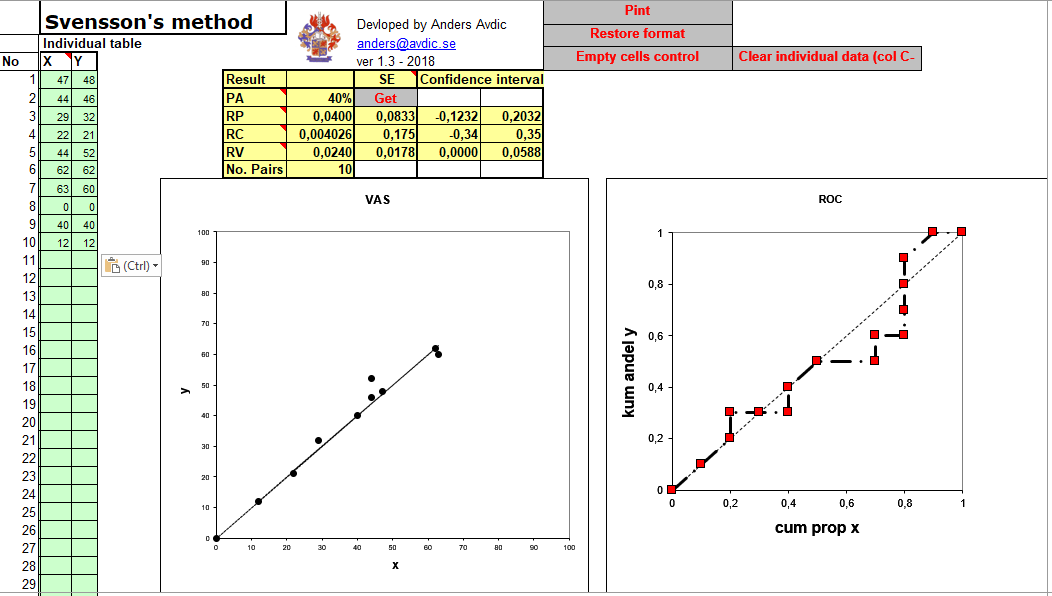


Figure 13. Total A-D, Upper Extremity inter-rater, day1_ scatterplot and ROC curve


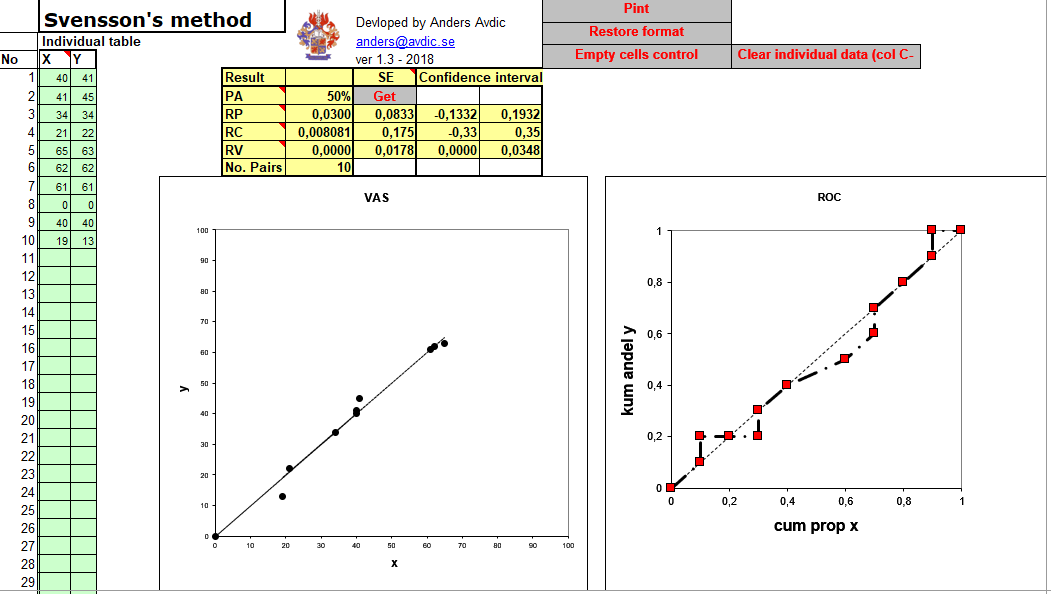
Figure 14. Total A-D, Upper Extremity inter-rater, day2_ scatterplot and ROC curve

### H scale Upper Extremity


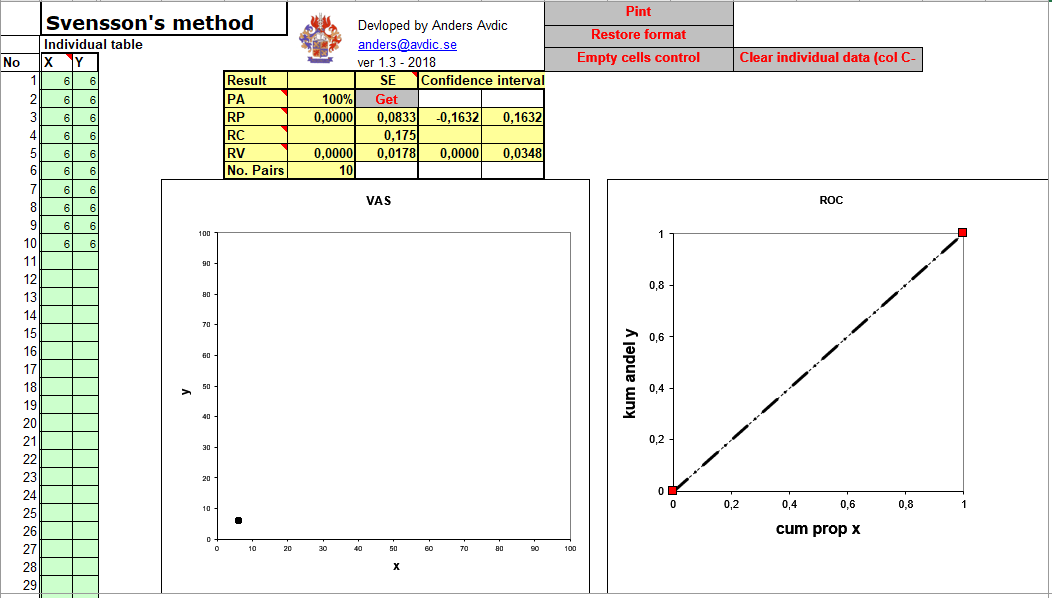
Figure 15. H scale, Upper Extremity inter-rater, day1_ scatterplot and ROC curve


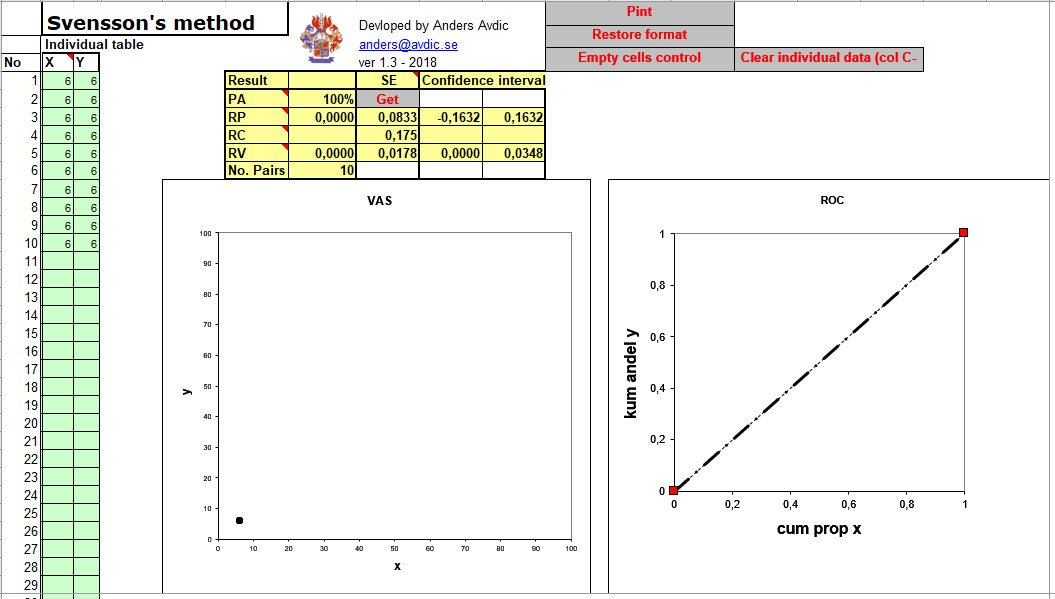
Figure 16. H scale, Upper Extremity inter-rater, day2_ scatterplot and ROC curve

### I scale Upper Extremity


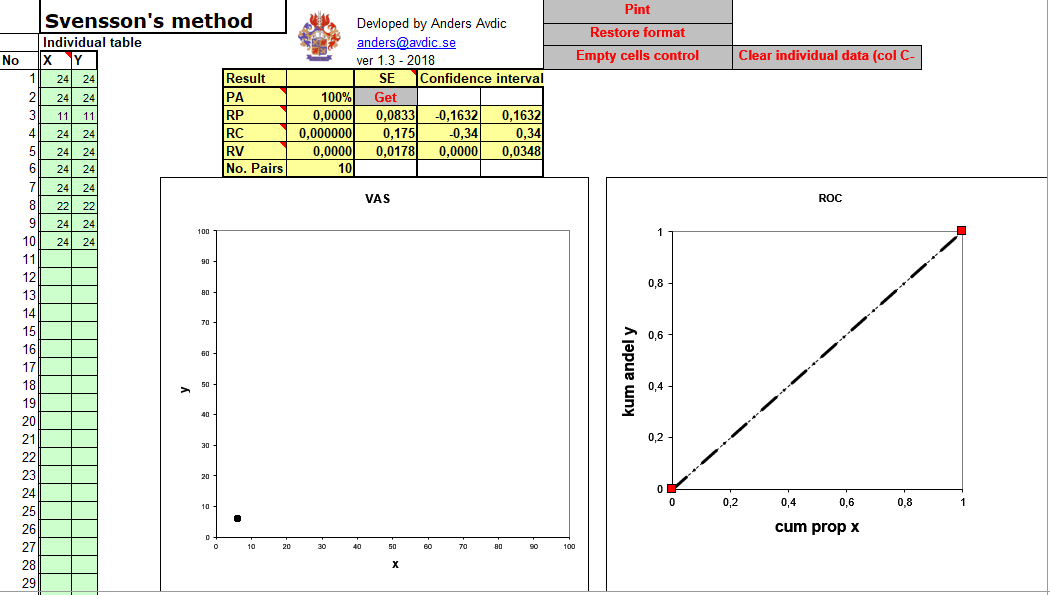
Figure 17. I scale, Upper Extremity inter-rater, day1_ scatterplot and ROC curve


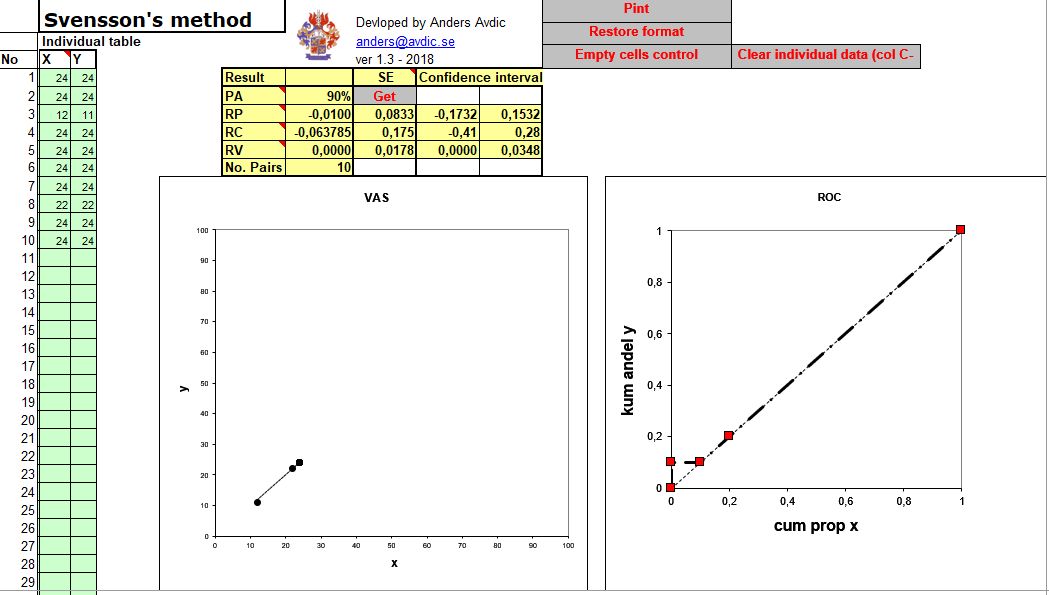
Figure 18. I scale, Upper Extremity inter-rater, day2_ scatterplot and ROC curve

### J scale Upper Extremity


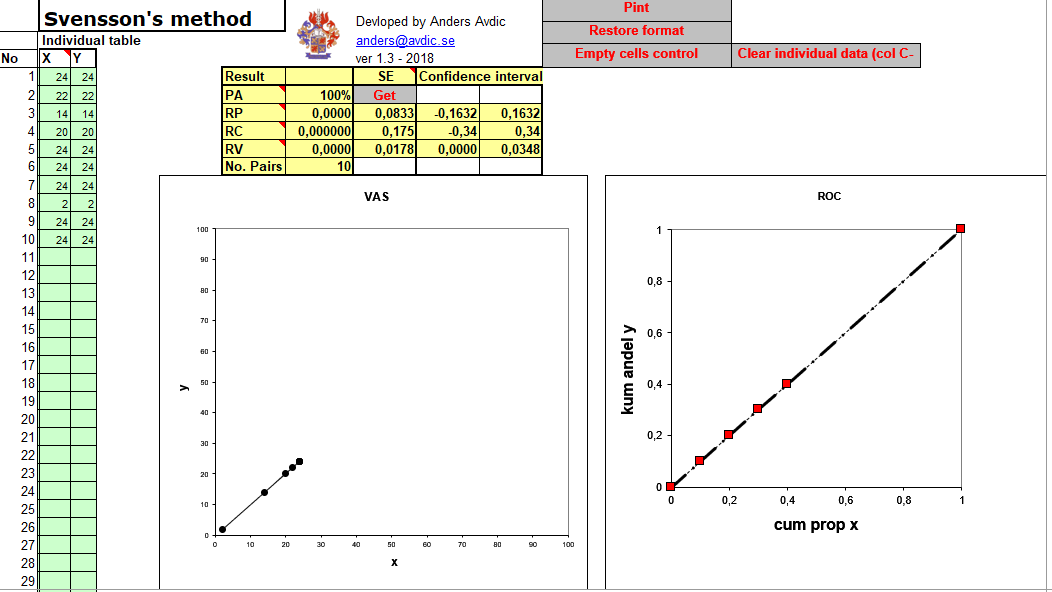
Figure 19. J scale, Upper Extremity inter-rater, day1_ scatterplot and ROC curve


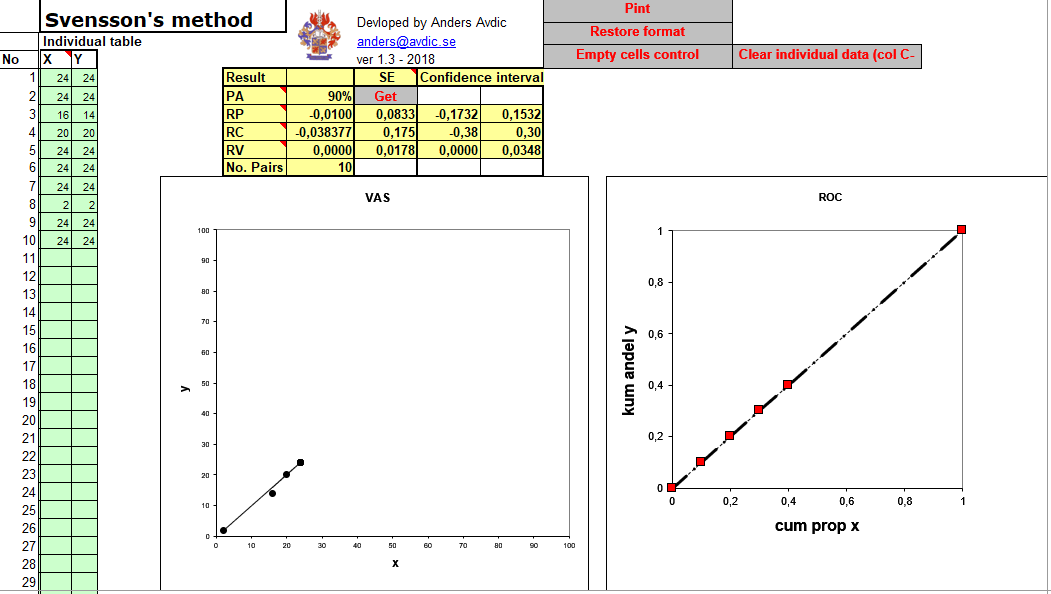
Figure 20. J scale, Upper Extremity inter-rater, day2_ scatterplot and ROC curve

### E scale Lower Extremity


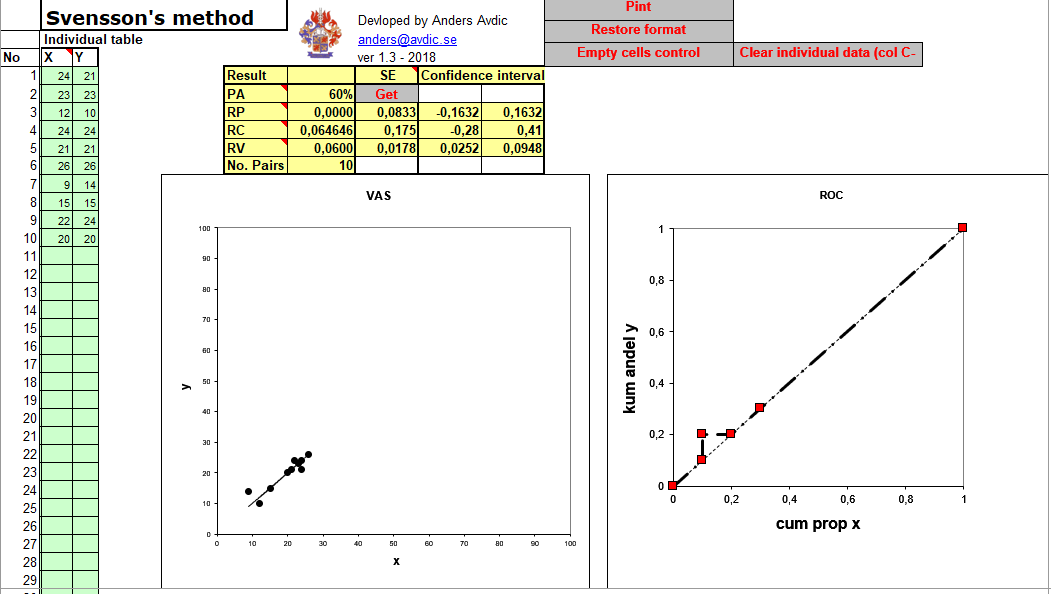
Figure 21. E scale, Lower Extremity inter-rater, day1_ scatterplot and ROC curve


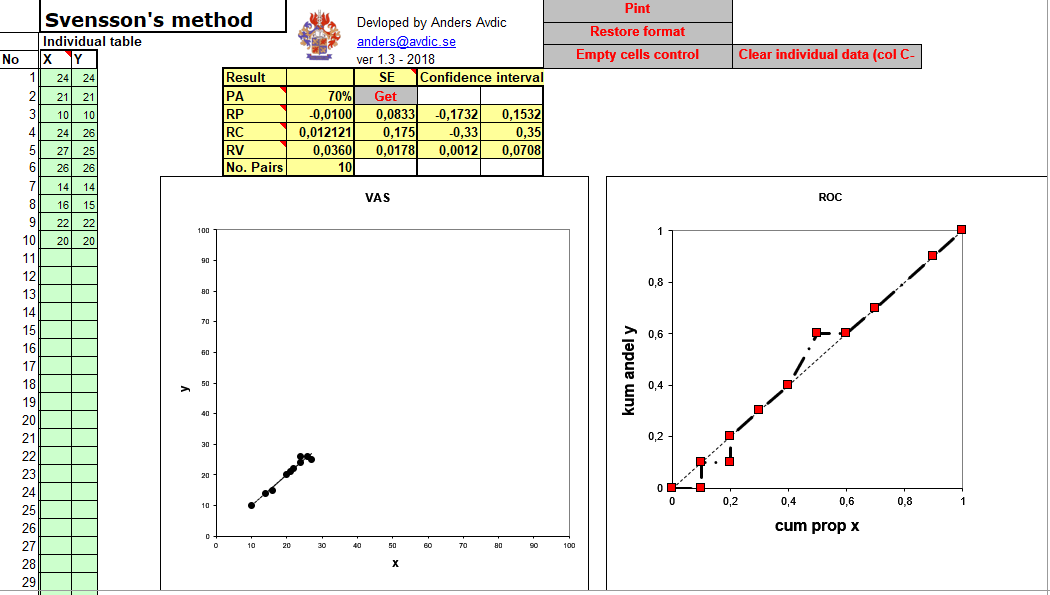
Figure 22. E scale, Lower Extremity inter-rater, day2_ scatterplot and ROC curve

### F scale Lower Extremity

This scale allows 7 possible values, i.e. from 0 to 6, hence the 11-category Svensson template was used. Below the results, the lowest percentage agreement being of 90%. Both the disagreement related measures had very good values as well:


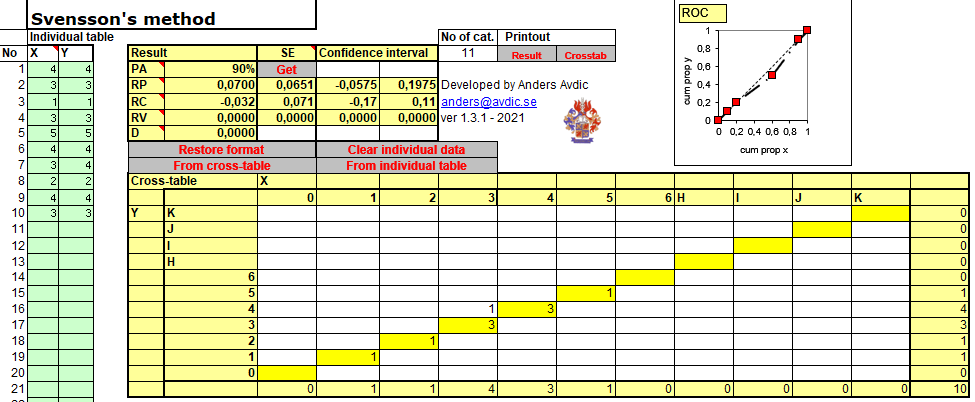
Figure 23. F scale, Lower Extremity inter-rater, day1_crosstab


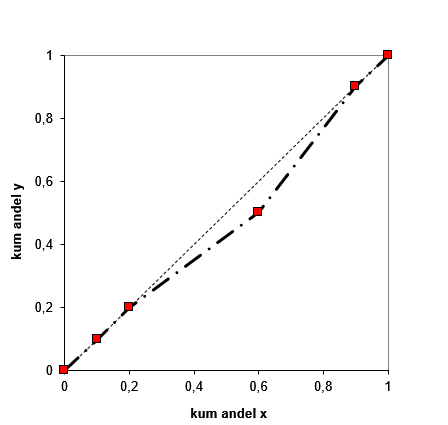


Figure 24. F scale, Lower Extremity inter-rater, day1_ROC curve


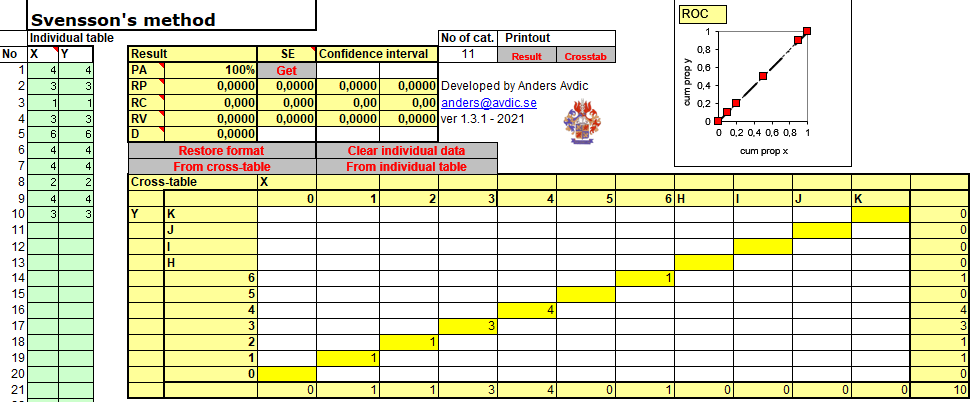
Figure 25. F scale, Lower Extremity inter-rater, day2_crosstab


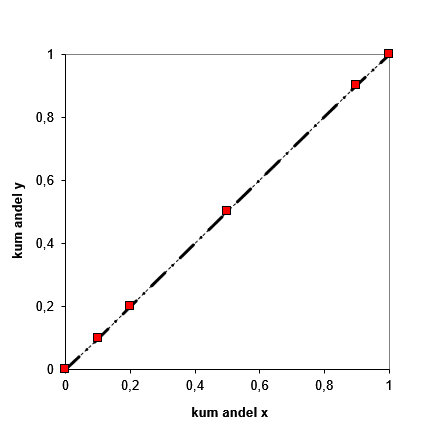


Figure 26. F scale, Lower Extremity inter-rater, day2_ROC curve

### Total E-F Lower Extremity (motor function)


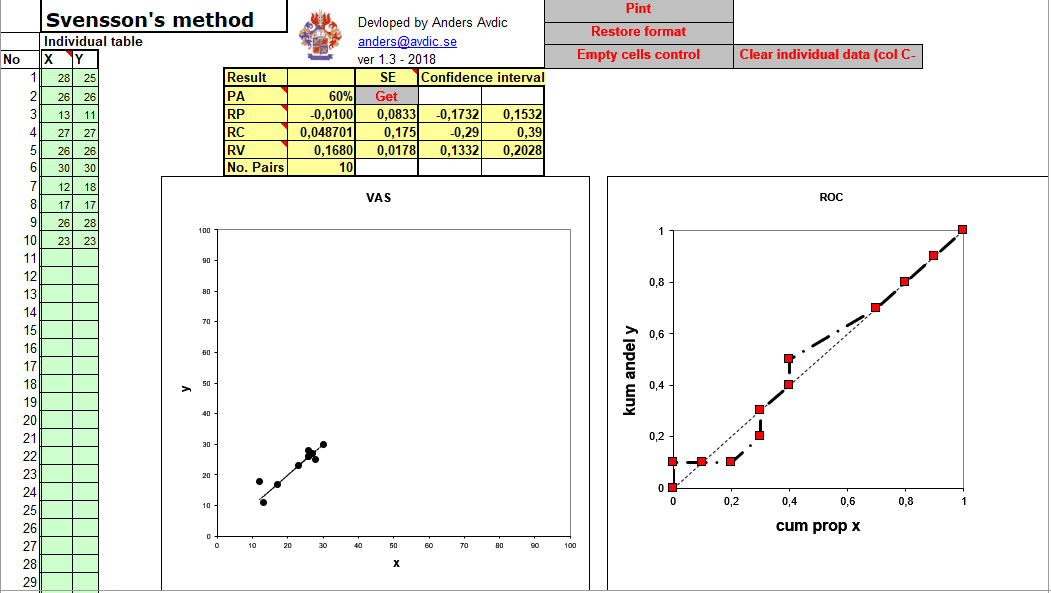
Figure 27. Total E-F, Lower Extremity inter-rater, day1_ scatterplot and ROC curve


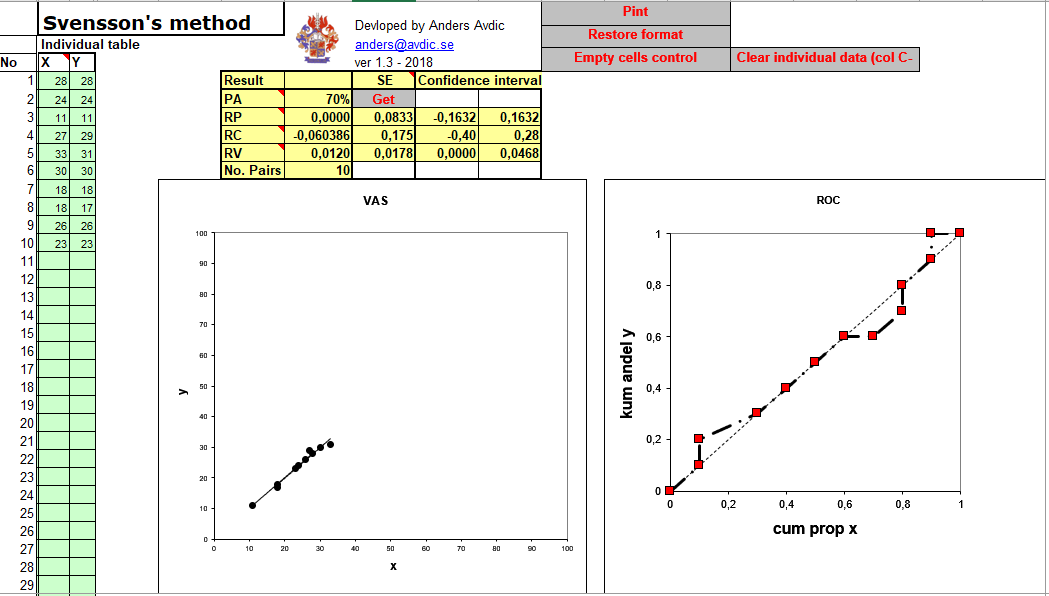
Figure 28. Total E-F, Lower Extremity inter-rater, day2_ scatterplot and ROC curve

### H scale Lower Extremity


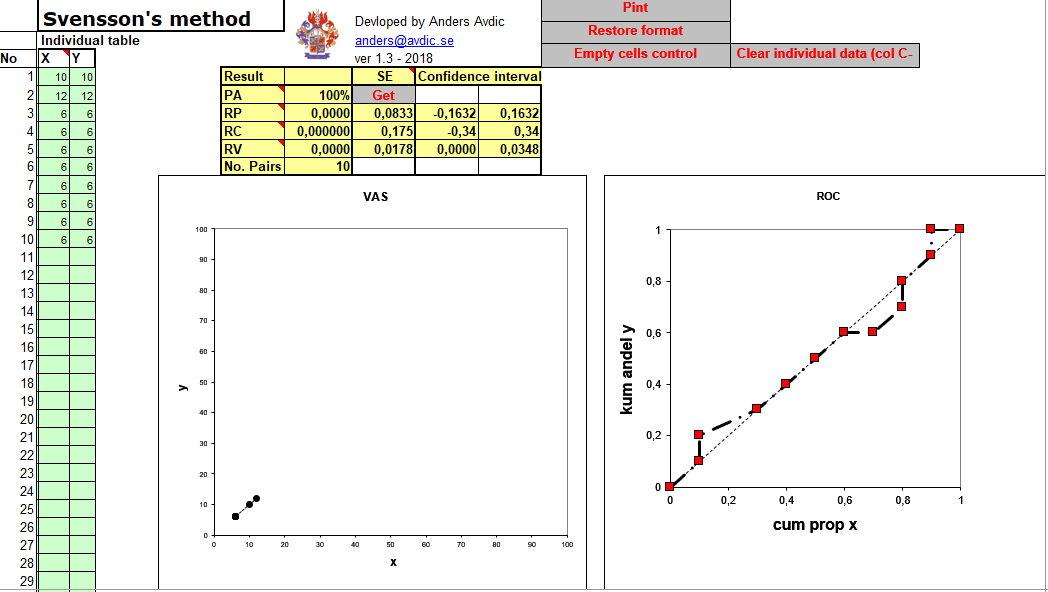
Figure 29. H scale, Lower Extremity inter-rater, day1_ scatterplot and ROC curve


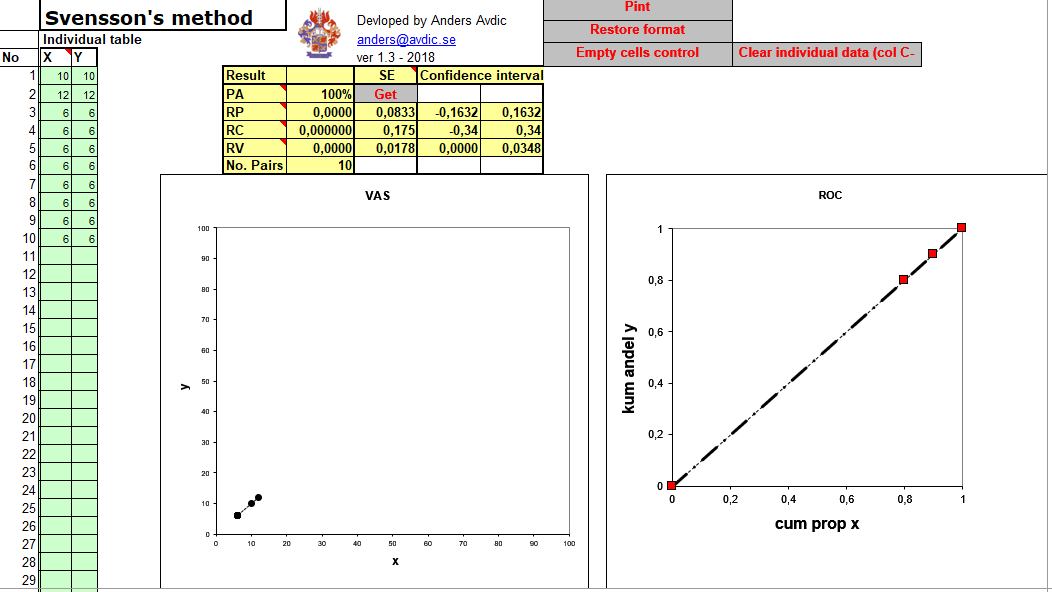
Figure 30. H scale, Lower Extremity inter-rater, day2_ scatterplot and ROC curve

### I scale Lower Extremity


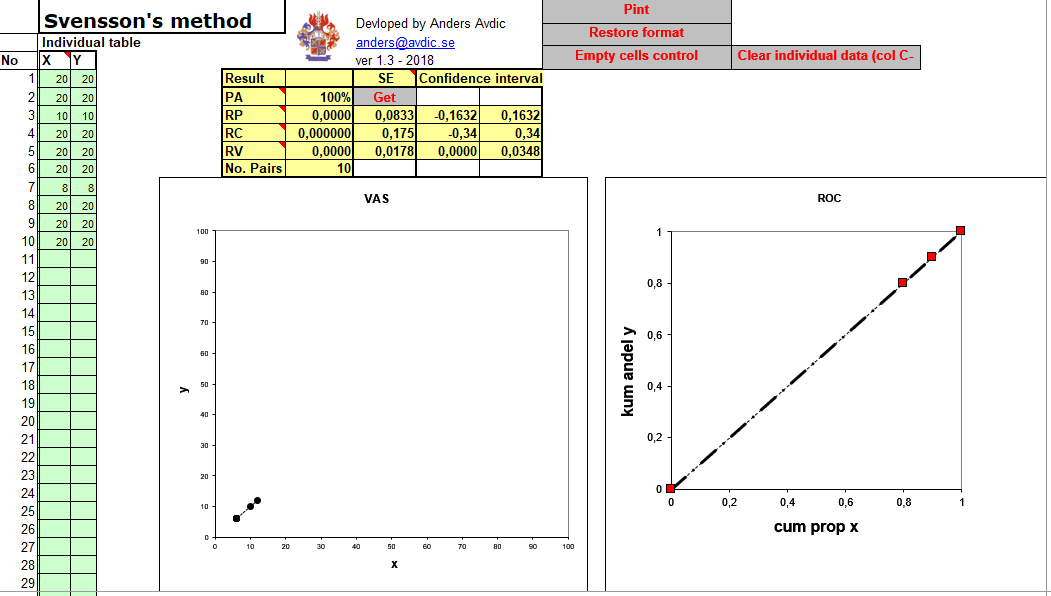
Figure 31. I scale, Lower Extremity inter-rater, day1_ scatterplot and ROC curve


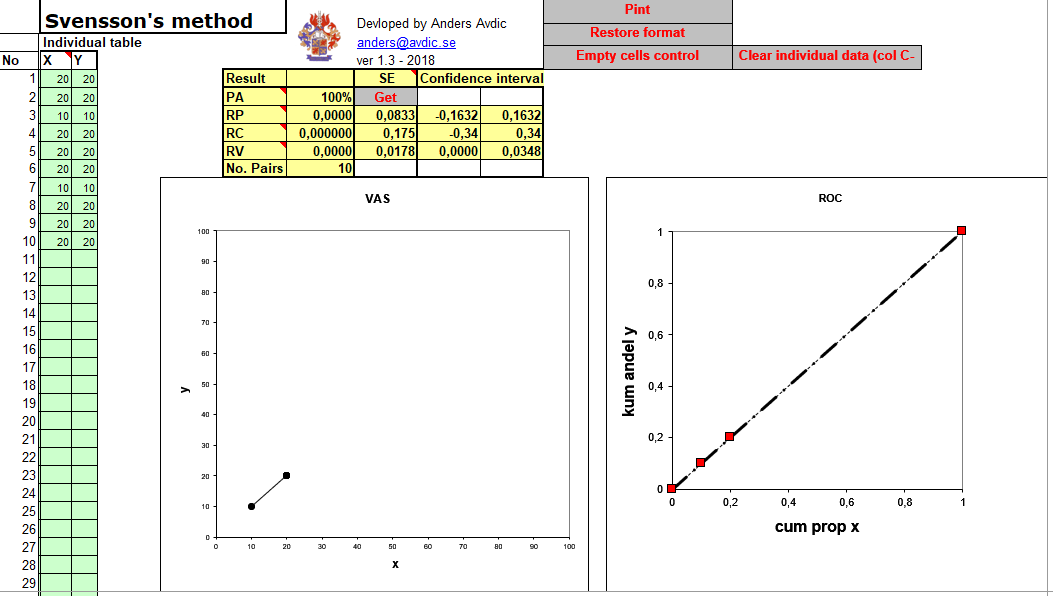
Figure 32. I scale, Lower Extremity inter-rater, day2_ scatterplot and ROC curve

### J scale Lower Extremity


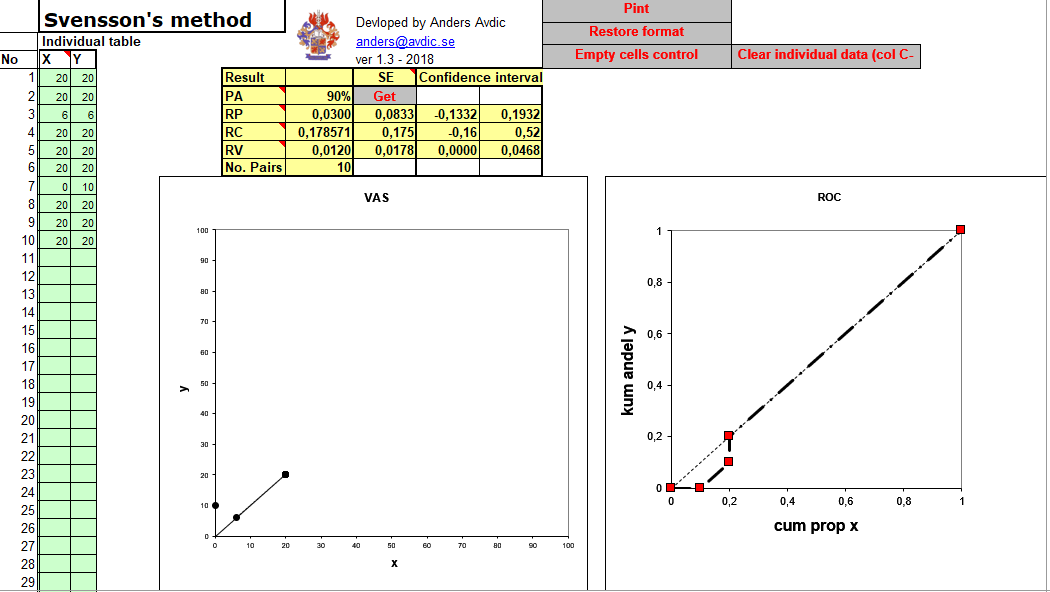
Figure 33. J scale, Lower Extremity inter-rater, day1_ scatterplot and ROC curve


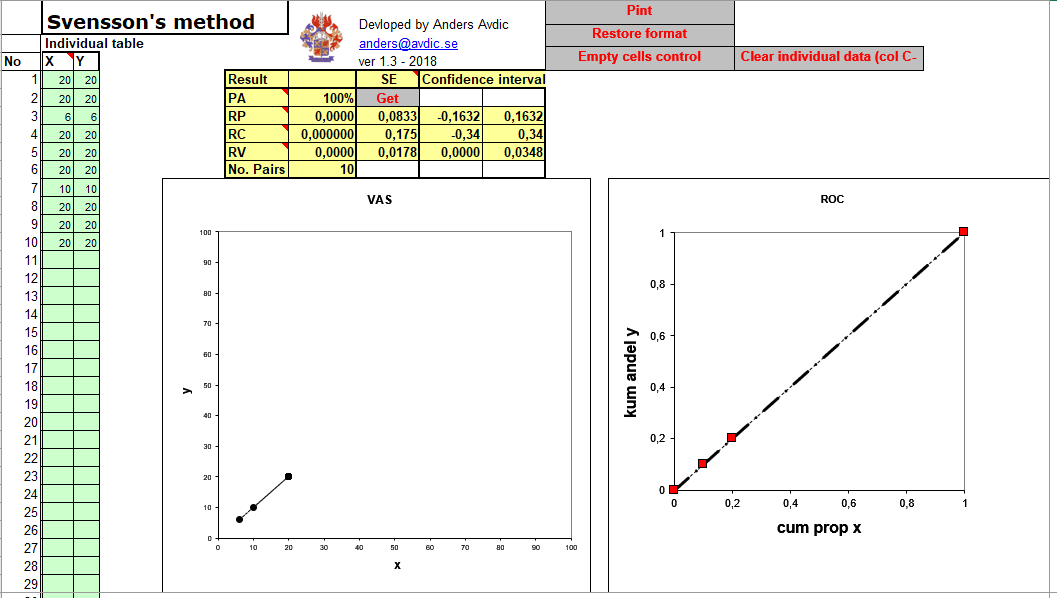
Figure 34. J scale, Lower Extremity inter-rater, day2_ scatterplot and ROC curve

## INTRA-rater Svensson’s results

The intra-rater Svensson’s method was done comparing values from day1 with values from day2, for each of the KTs, therefore the method was applied two times (each with the 10 patients), for each of the FMA scale parts. The aim here is to prove that the scale is consistently interpreted by the same person/KT, irrespective of the moments (as long as such moments of evaluation are close one from the other). Here as well, the templates used were 11 categories template for B, D and F scales, and VAS scatterplot for the rest of them.

### A scale Upper Extremity


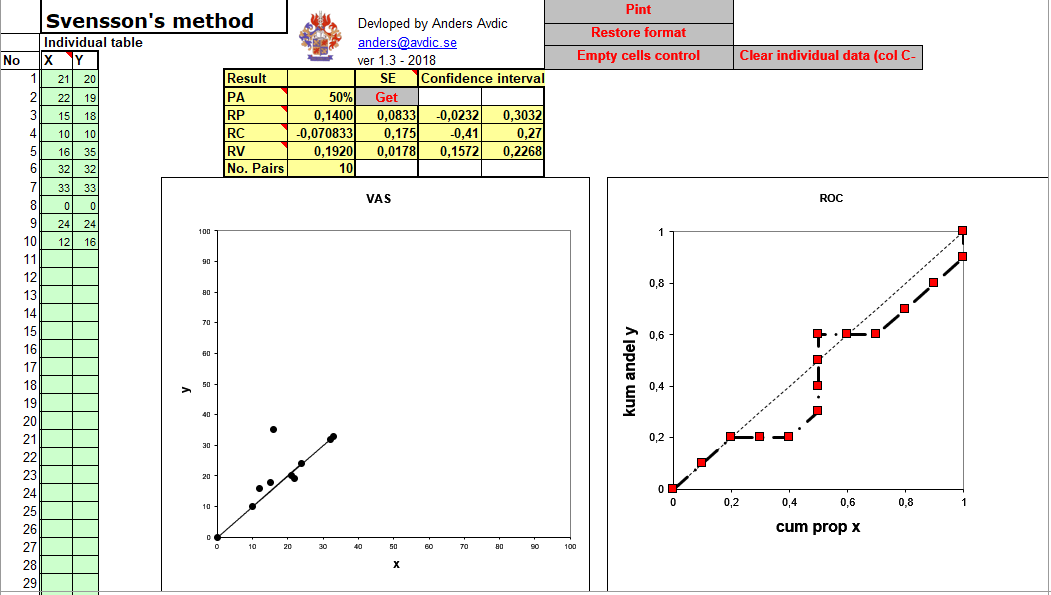
Figure 35. A scale, Upper Extremity intra-rater, KT1_ scatterplot and ROC curve


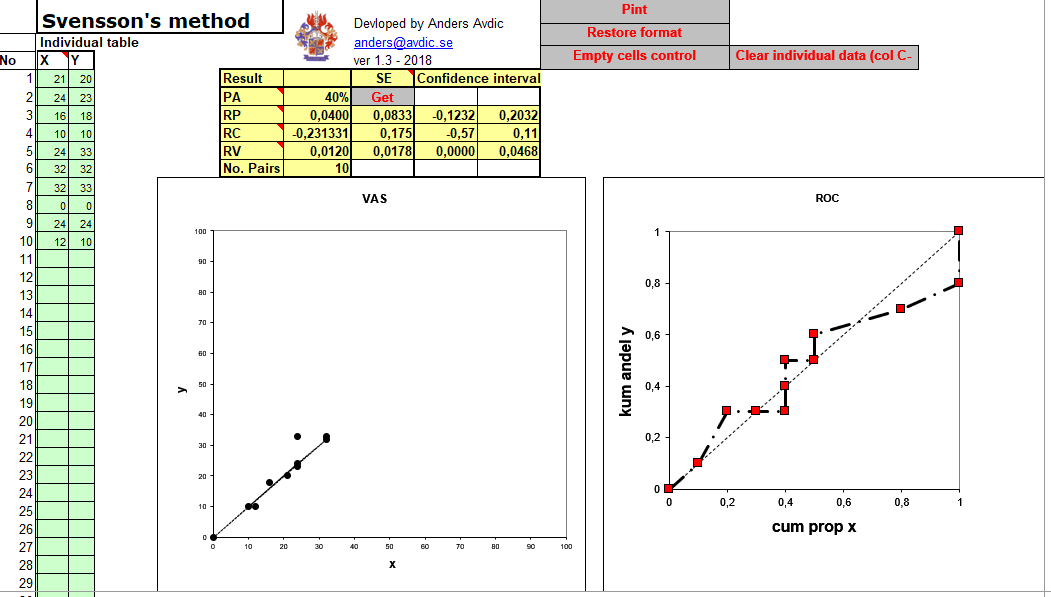
Figure 36. A scale, Upper Extremity intra-rater, KT2_ scatterplot and ROC curve

### B scale Upper Extremity

Below the results for this scale. Both the Percentage Agreement values are at least at 80% level, while in one case out of four, the Relative Concentration had a slightly higher than 0.1 upper threshold value:


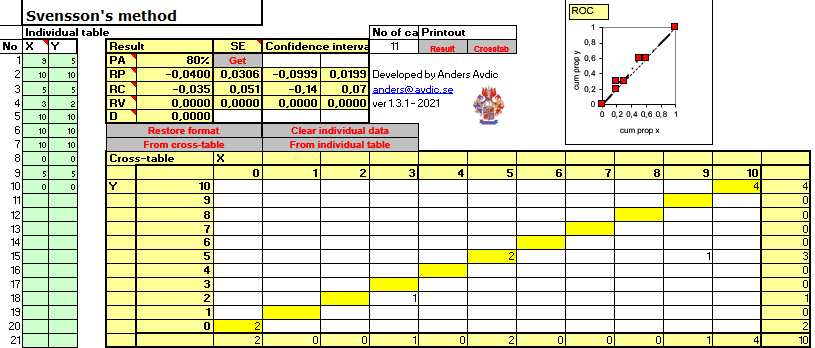
Figure 37. B scale, Upper Extremity intra-rater, KT1_crosstab
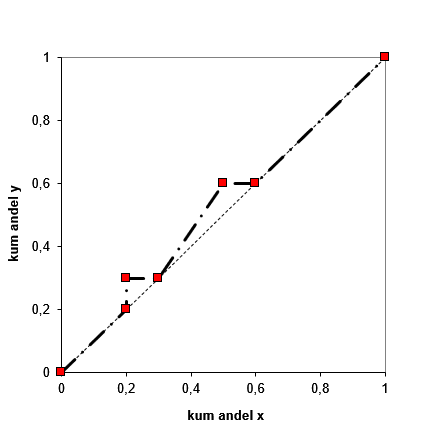


Figure 38. B scale, Upper Extremity intra-rater, KT1_ROC curve


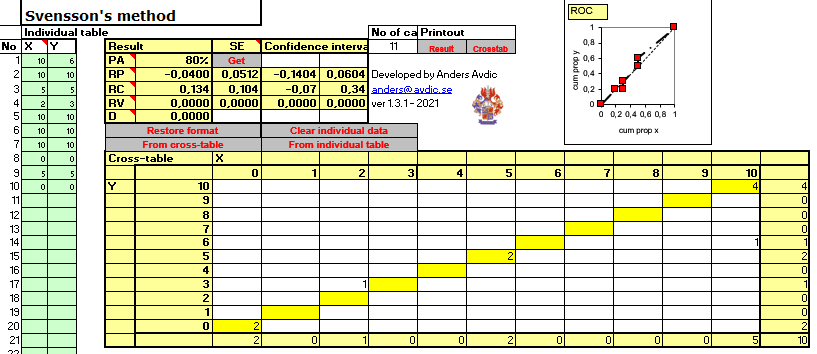
Figure 39. B scale, Upper Extremity intra-rater, KT2_crosstab


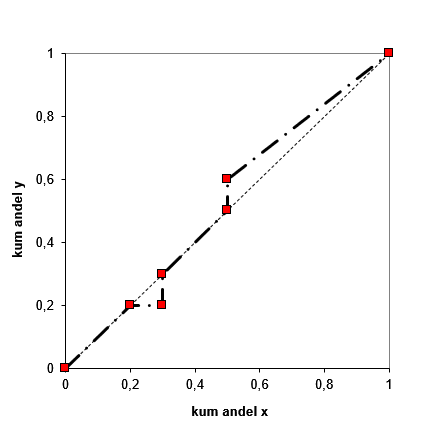


Figure 40. B scale, Upper Extremity intra-rater, KT2_ROC curve

### C scale Upper Extremity


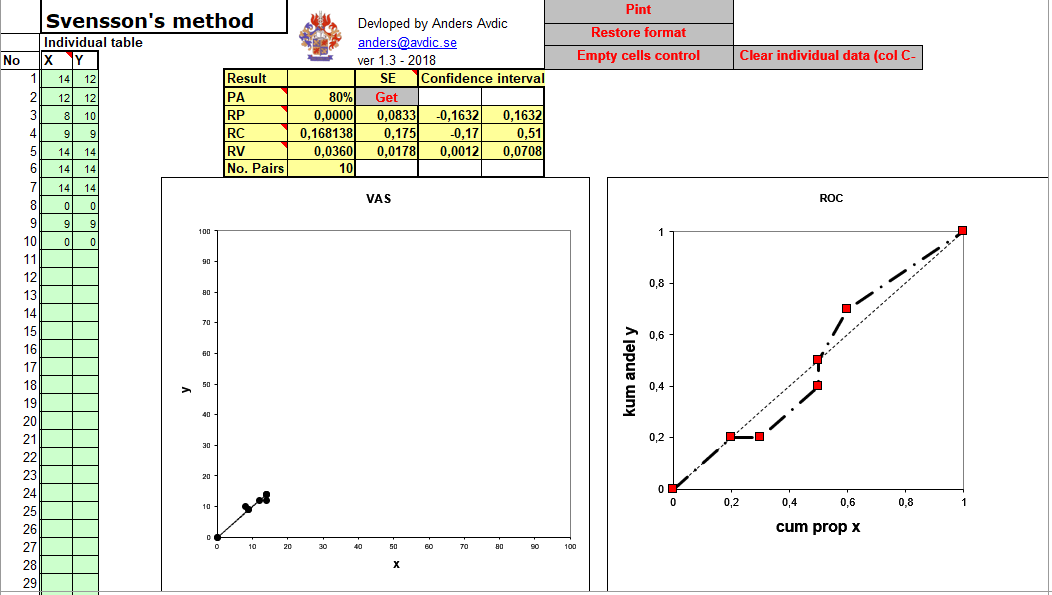
Figure 41. C scale, Upper Extremity intra-rater, KT1_ scatterplot and ROC curve


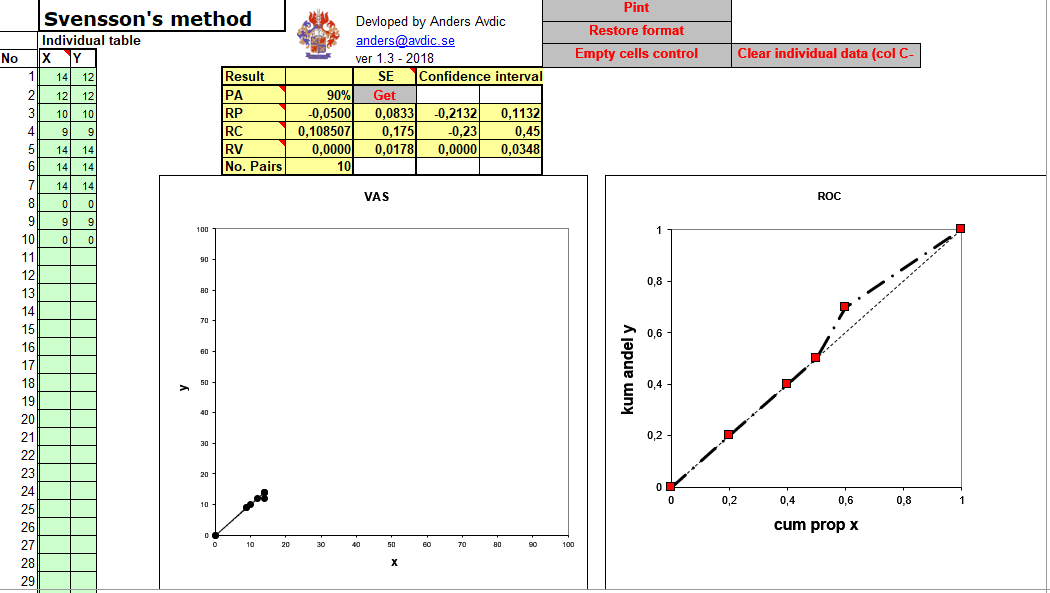
Figure 42. C scale, Upper Extremity intra-rater, KT2_ scatterplot and ROC curve

### D scale Upper Extremity

The results show a PA of 70-80%. As per the Relative Position, there was a comparison with a value slightly higher than the 0.1 limit:


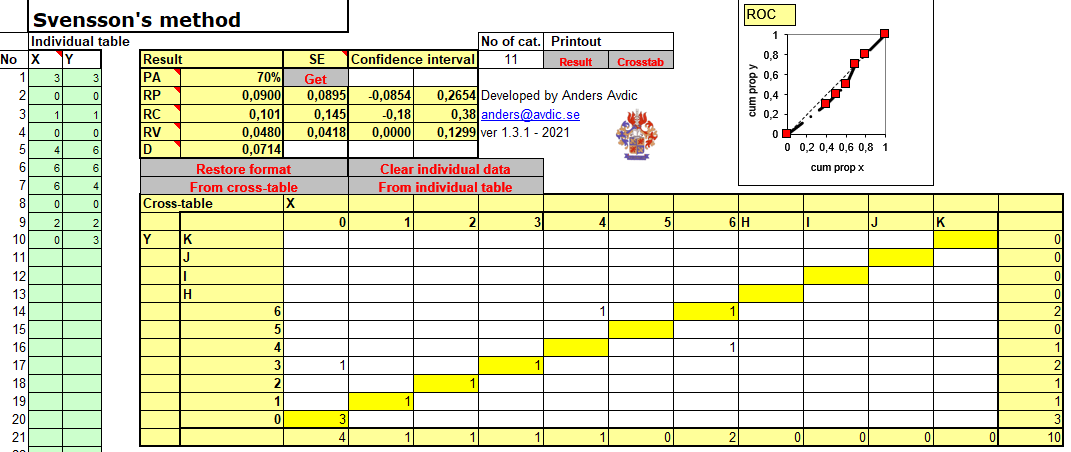
Figure 43. D scale, Upper Extremity intra-rater, KT1_crosstab


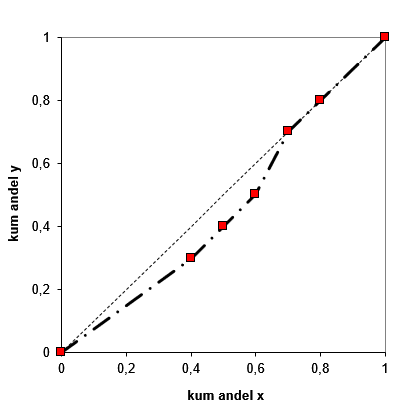


Figure 44. D scale, Upper Extremity intra-rater, KT1_ROC curve


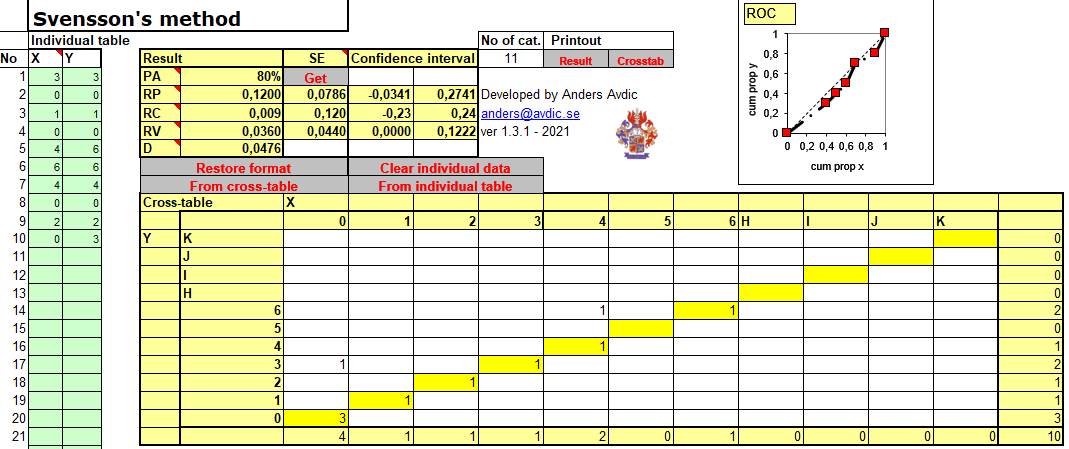
Figure 45. D scale, Upper Extremity intra-rater, KT2_crosstab


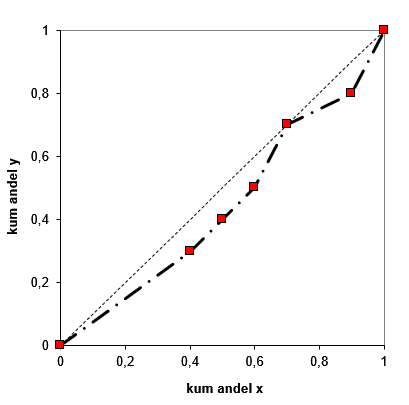


Figure 46. D scale, Upper Extremity intra-rater, KT2_ROC curve

### Total A-D Upper Extremity (motor function)


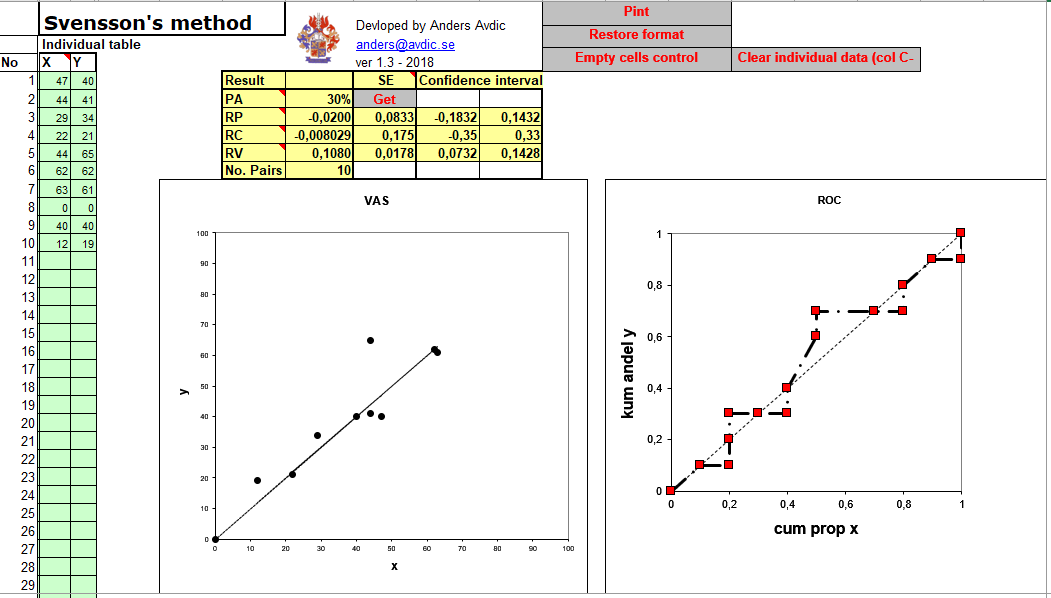
Figure 47. Total A-D, Upper Extremity intra-rater, KT1_ scatterplot and ROC curve


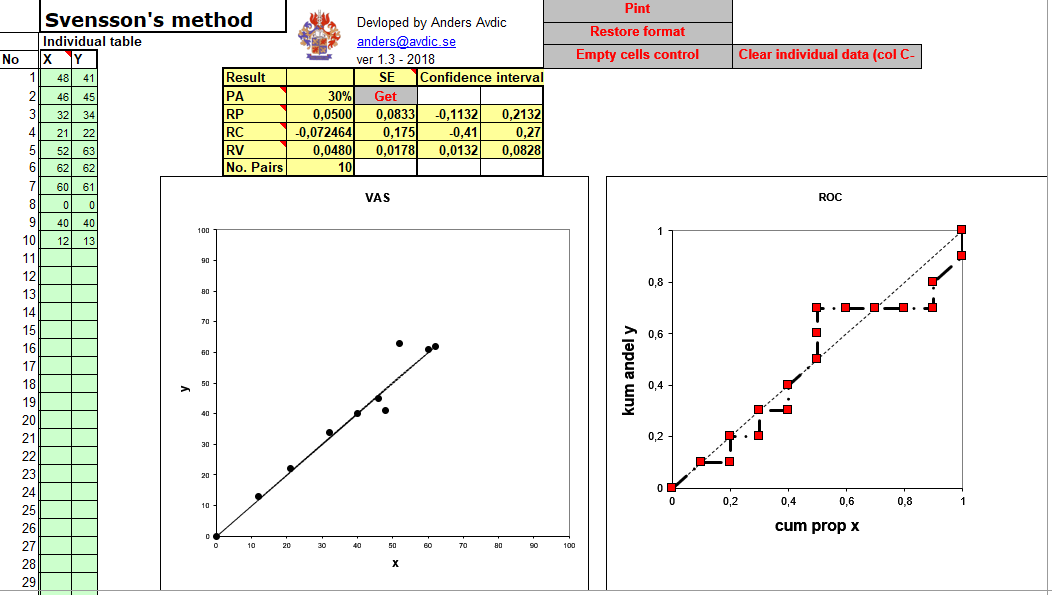
Figure 48. Total A-D, Upper Extremity intra-rater, KT2_ scatterplot and ROC curve

### H scale Upper Extremity


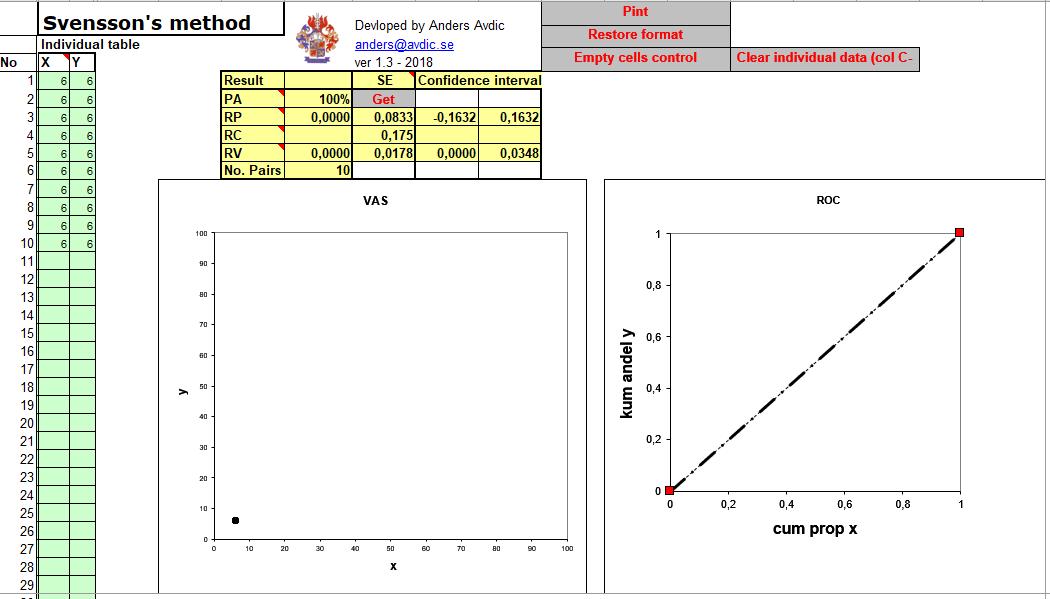
Figure 49. H scale, Upper Extremity intra-rater, KT1_ scatterplot and ROC curve


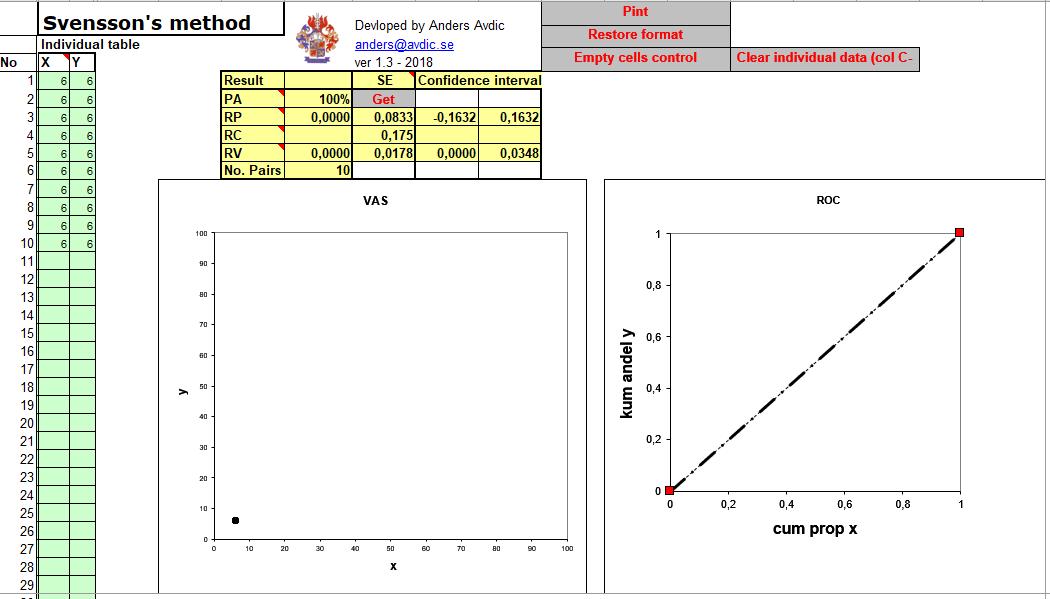
Figure 50. H scale, Upper Extremity intra-rater, KT2_ scatterplot and ROC curve

### I scale Upper Extremity


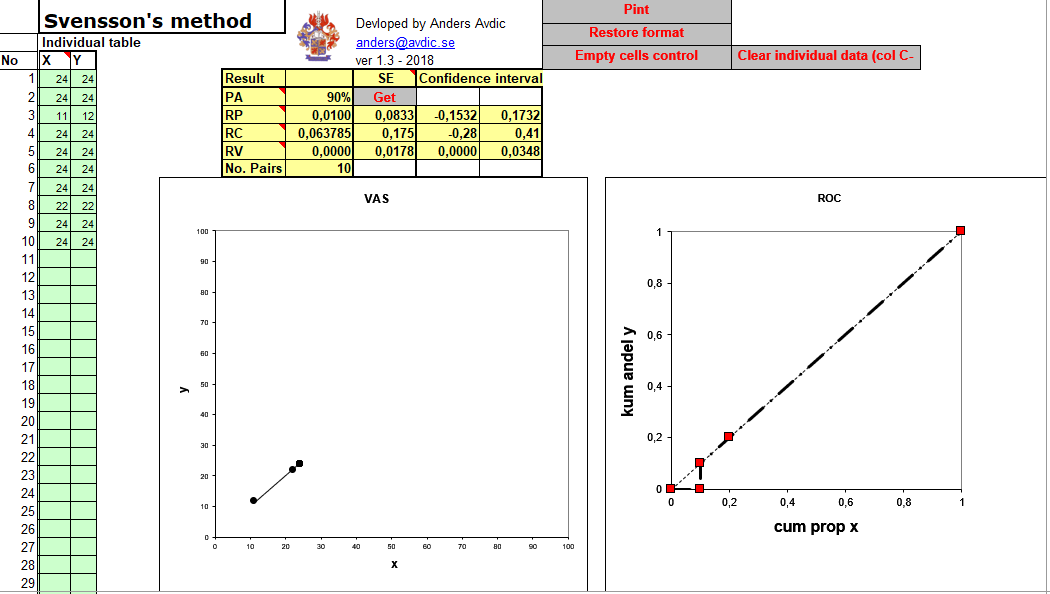
Figure 51. I scale, Upper Extremity intra-rater, KT1_ scatterplot and ROC curve


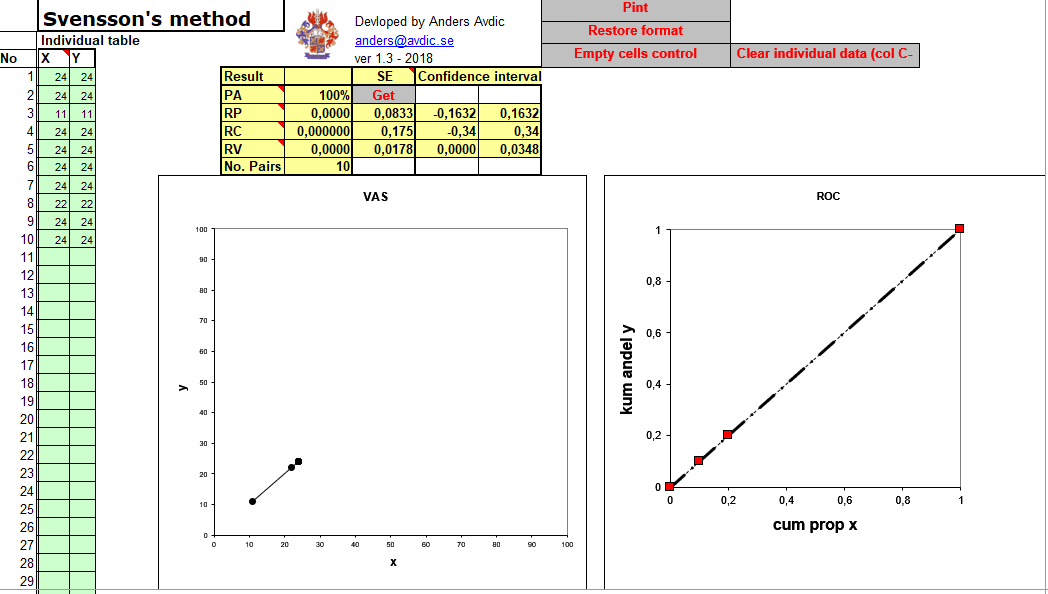
Figure 52. I scale, Upper Extremity intra-rater, KT2_ scatterplot and ROC curve

### J scale Upper Extremity


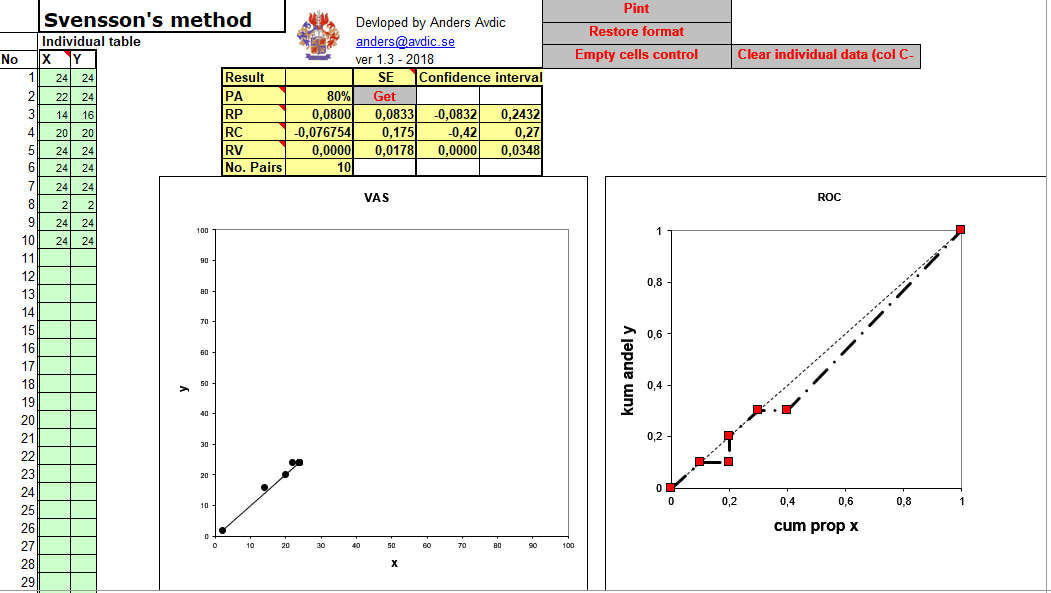
Figure 53. J scale, Upper Extremity intra-rater, KT1_ scatterplot and ROC curve


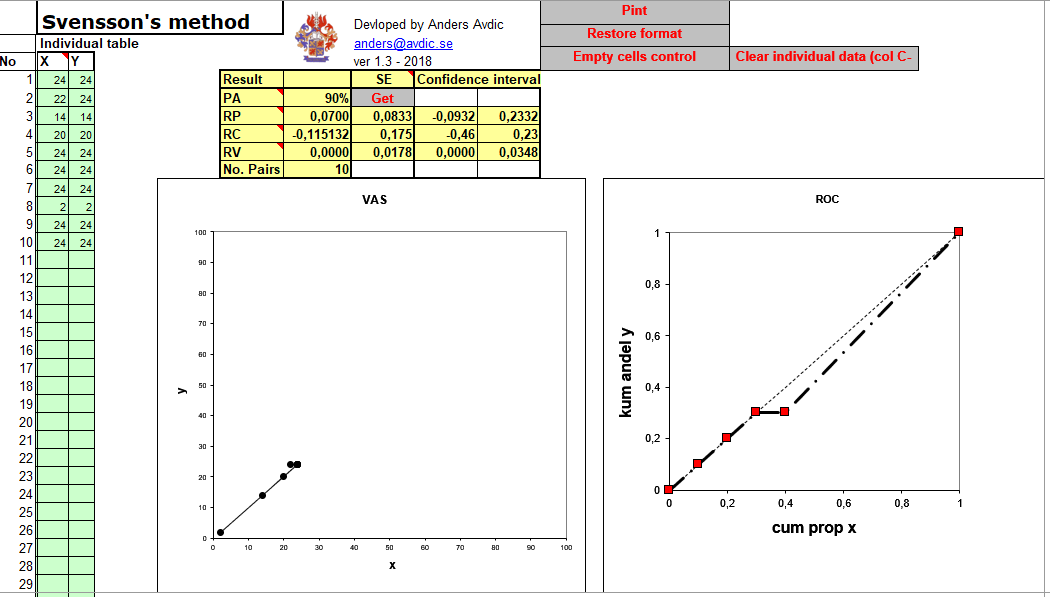
Figure 54. J scale, Upper Extremity intra-rater, KT2_ scatterplot and ROC curve

### E scale Lower Extremity


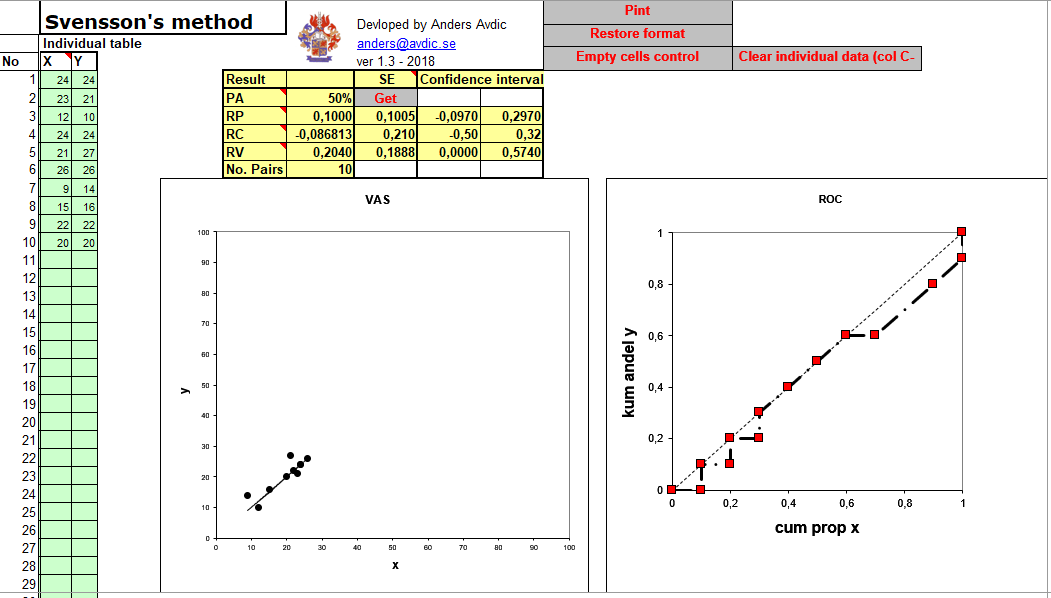
Figure 55. E scale, Lower Extremity intra-rater, KT1_ scatterplot and ROC curve


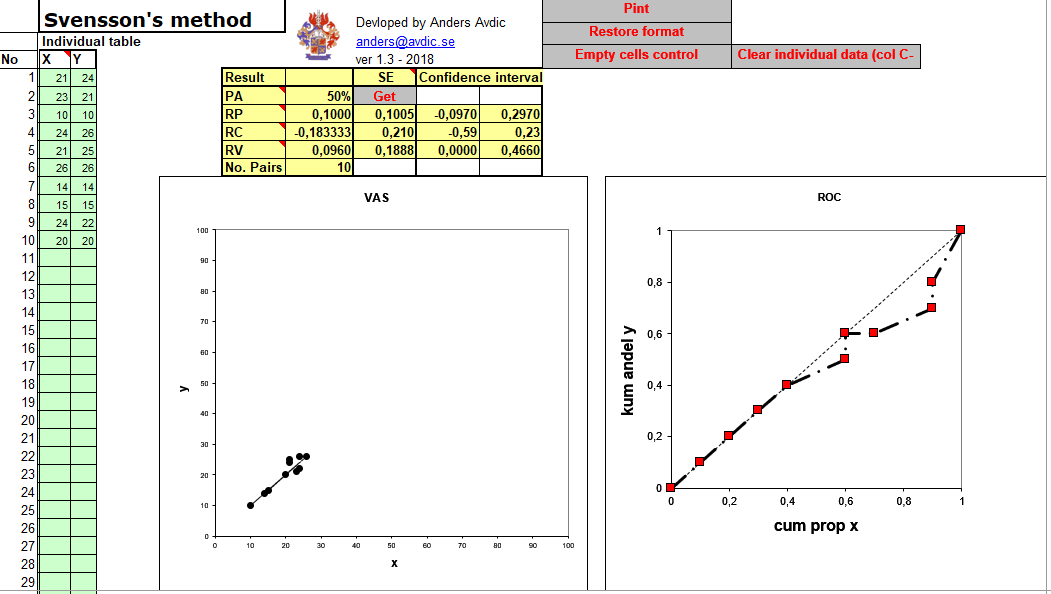
Figure 56. E scale, Lower Extremity intra-rater, KT2_ scatterplot and ROC curve

### F scale Lower Extremity

Below the results for F scale (Lower Extremity). The results are at least at 80% percentage agreement level, below details. The disagreement related measure are well within the interval considered as satisfactory:


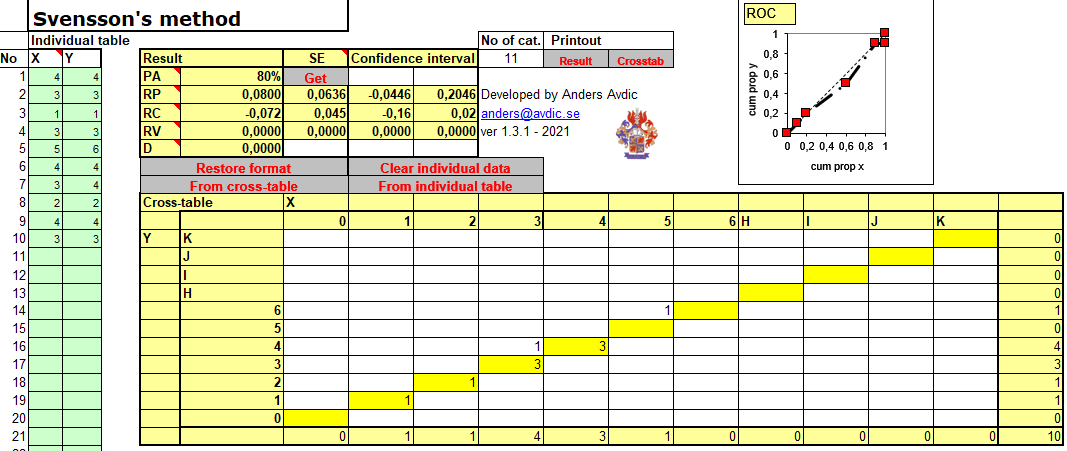
Figure 57. F scale, Lower Extremity intra-rater, KT1_crosstab


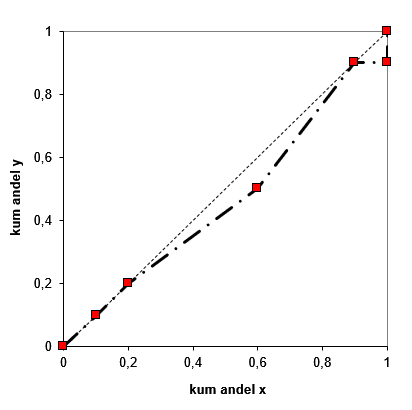


Figure 58. F scale, Lower Extremity intra-rater, KT1_ROC curve


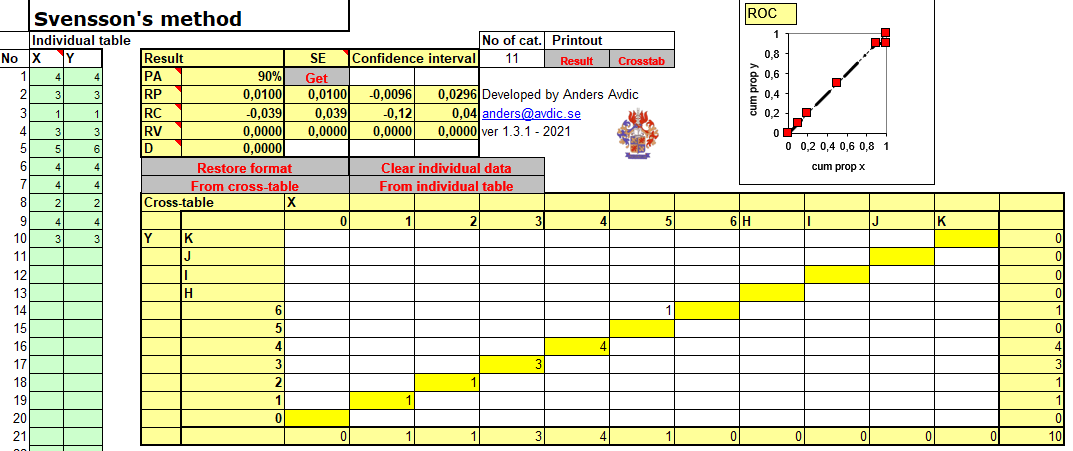
Figure 59. F scale, Lower Extremity intra-rater, KT2_crosstab


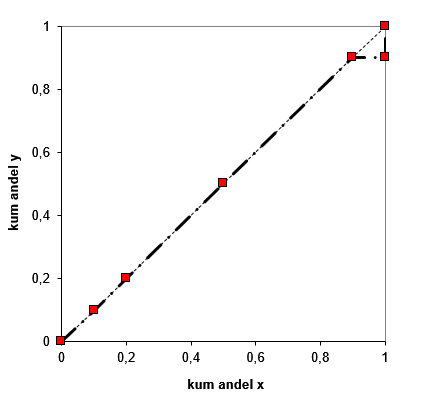


Figure 60. F scale, Lower Extremity intra-rater, KT2_ROC curve

### Total E-F Lower Extremity (motor function)


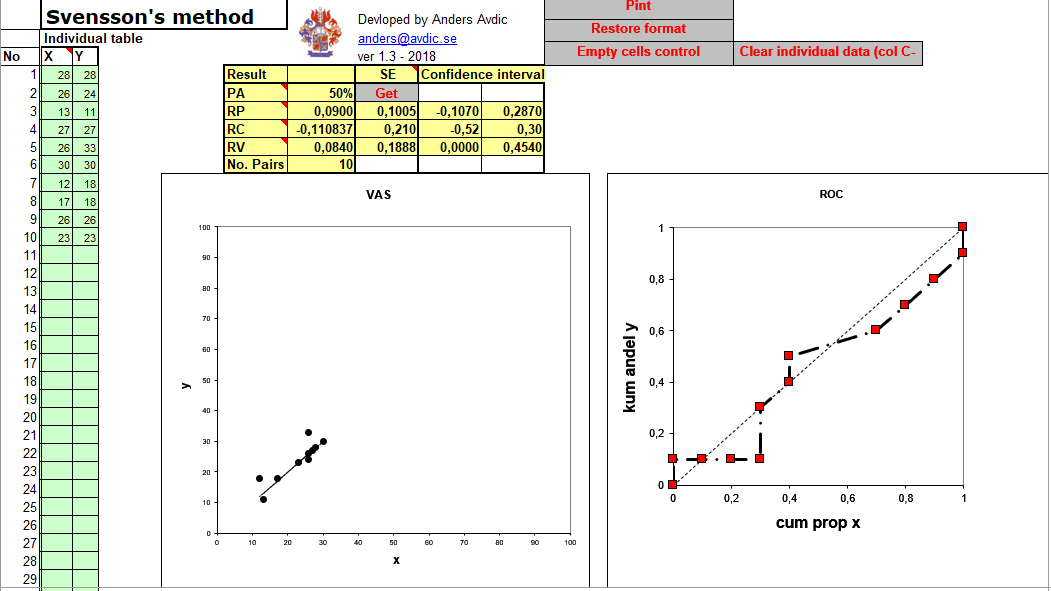
Figure 61. Total E-F, Lower Extremity intra-rater, KT1_ scatterplot and ROC curve


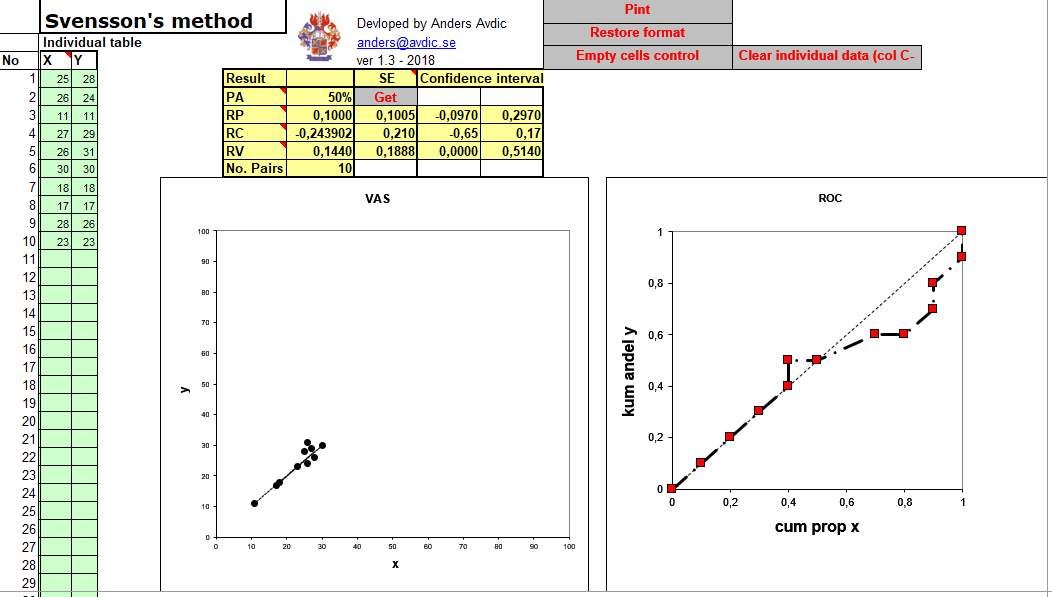
Figure 62. Total E-F, Lower Extremity intra-rater, KT2_ scatterplot and ROC curve

### H scale Lower Extremity


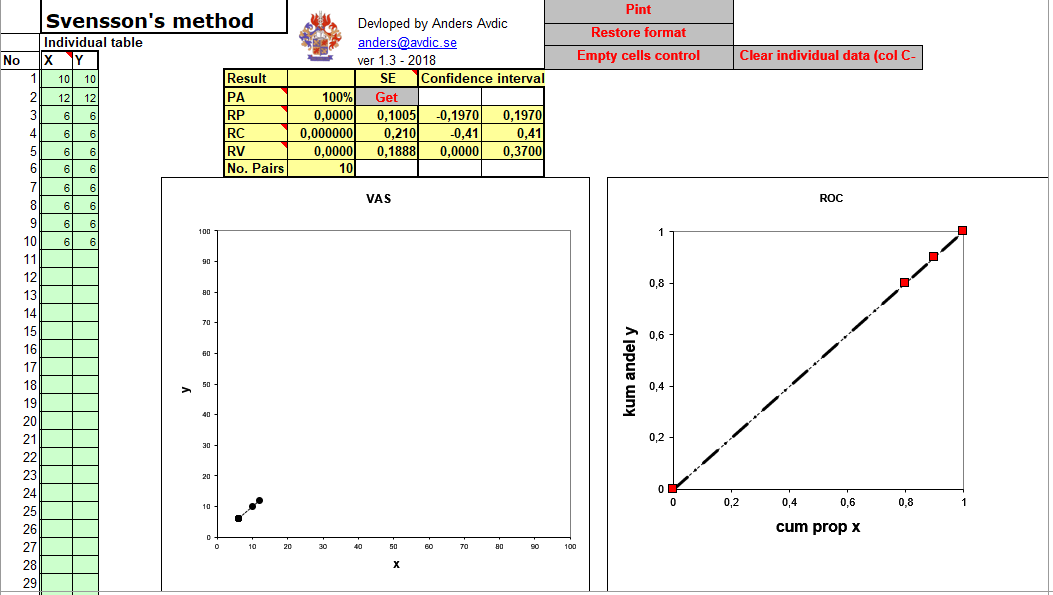
Figure 63. H scale, Lower Extremity intra-rater, KT1_ scatterplot and ROC curve


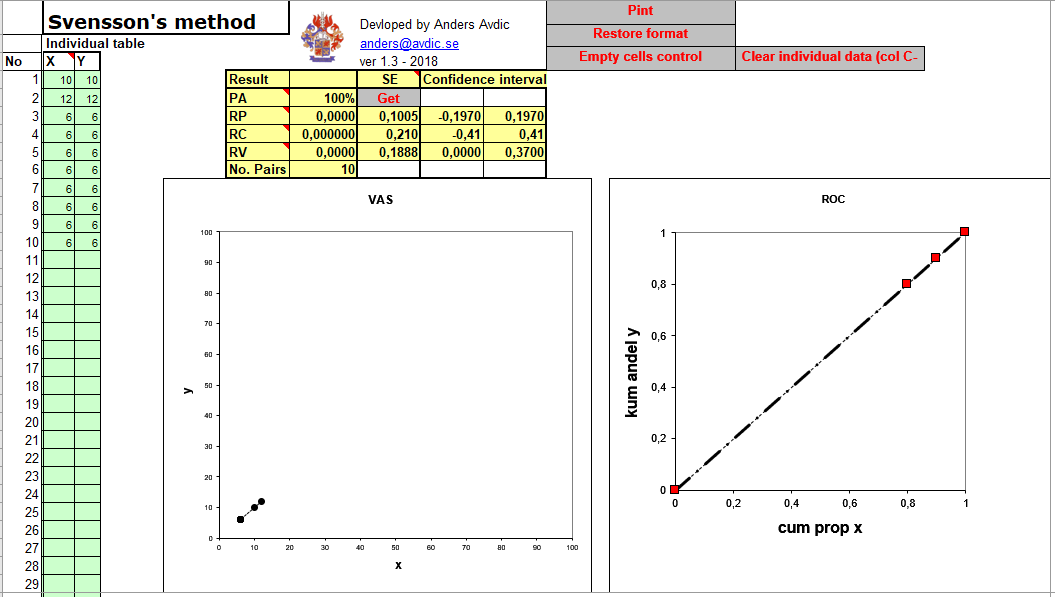
Figure 64. H scale, Lower Extremity intra-rater, KT2_ scatterplot and ROC curve

### I scale Lower Extremity


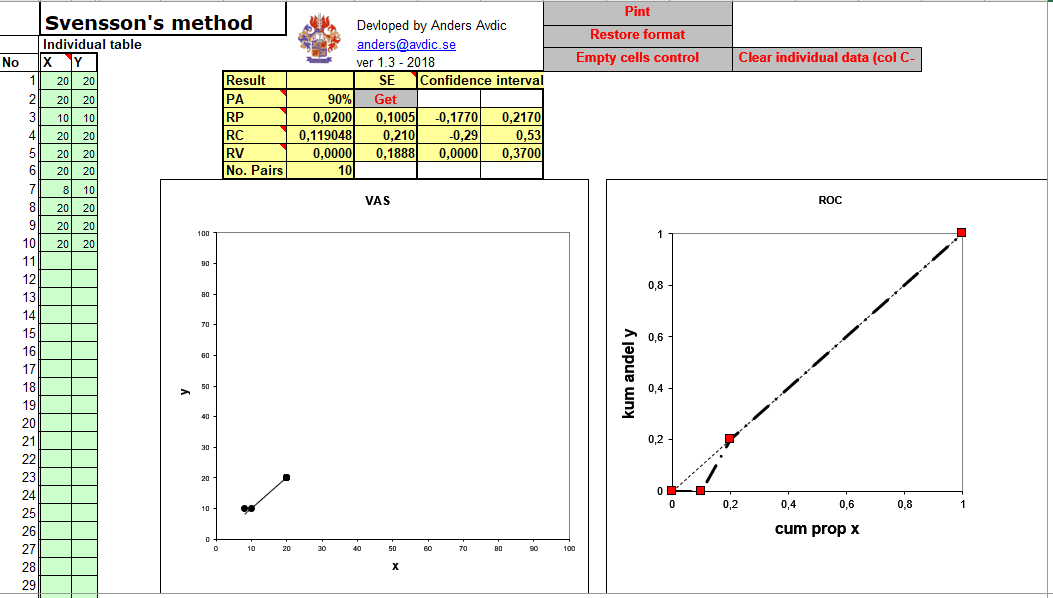
Figure 65. I scale, Lower Extremity intra-rater, KT1_ scatterplot and ROC curve


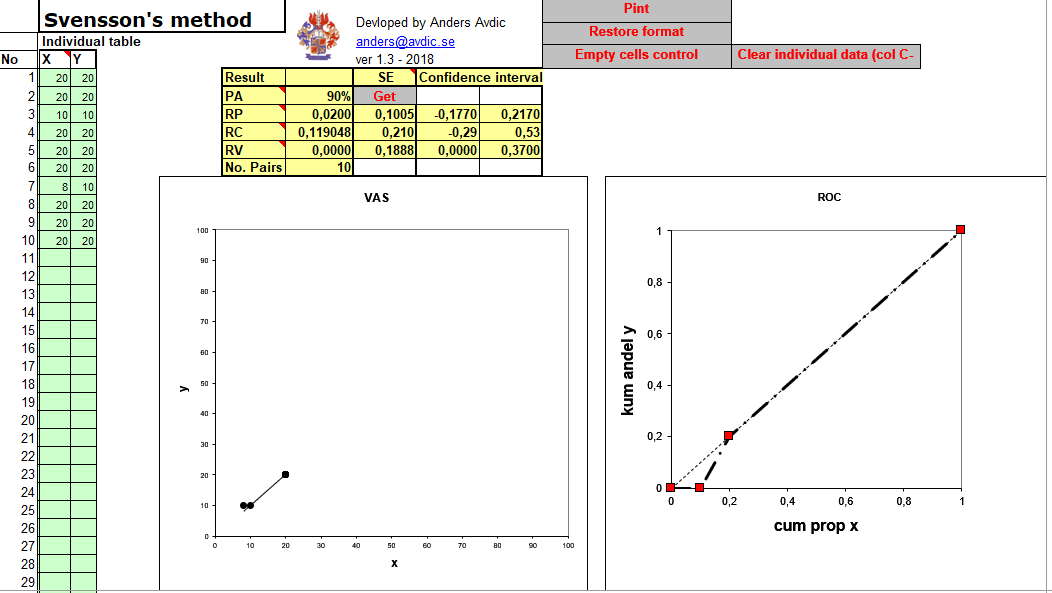
Figure 66. I scale, Lower Extremity intra-rater, KT2_ scatterplot and ROC curve

### J scale Lower Extremity


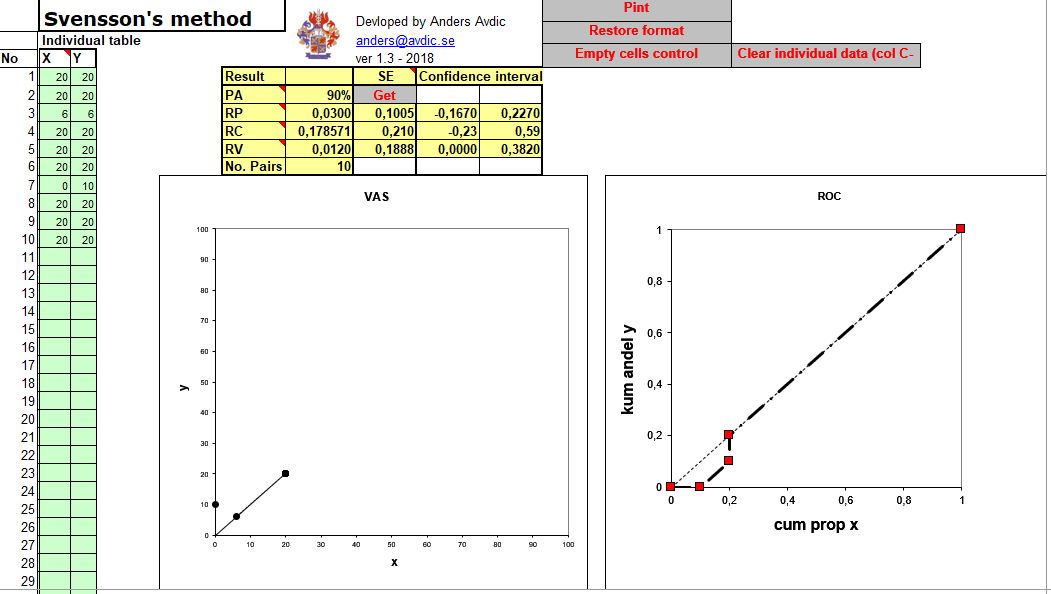
Figure 67. J scale, Lower Extremity intra-rater, KT1_ scatterplot and ROC curve


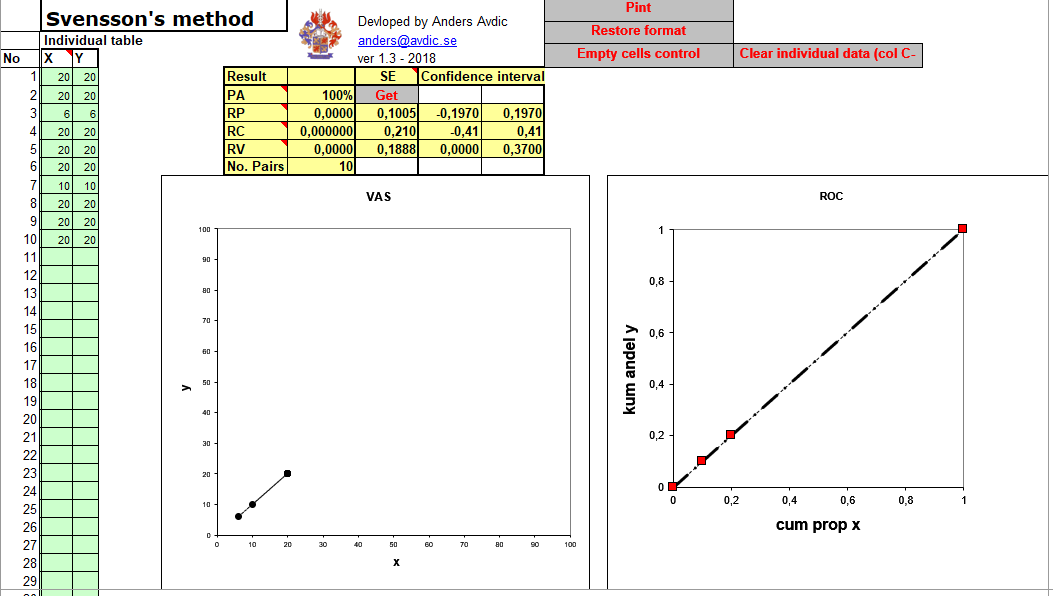
Figure 68. J scale, Lower Extremity intra-rater, KT2_ scatterplot and ROC curve

## Overall Svensson’s results table

The inter-rater Svensson’s method was done comparing values from KT1 with values from KT2 for each of the two days within the evaluation, therefore the method was applied two times (each with the 10 patients), for each of the FMA scale parts. The aim here is to prove that the interpretation of the scale is objective enough, that is, it is independent of whether it is evaluated by one KT or the other. The templates used were 11 categories template for B, D and F scales (as they allow maximum of 11 possible levels), and VAS scatterplot for the rest of them.

The intra-rater Svensson’s method was done comparing values from day1 with values from day2, for each of the KTs, therefore the method was applied two times (each with the 10 patients), for each of the FMA scale parts. The aim here is to prove that the scale is consistently interpreted by the same person/KT, irrespective of the moments (as long as such moments of evaluation are close one from the other). Here as well, the templates used were 11 categories template for B, D and F scales (as they allow maximum of 11 possible levels), and VAS scatterplot for the rest of them.

Svensson’s evaluation method was applied 56 times (including hence totals for motor functions), 28 times for inter-rater, and 28 times for intra-rater. We obtained good results also given the context (10 pairs of data). Below the results in both synthetic (averages – excluding totals) and detailed form (for each scale):

- **Percentage Agreement (PA)** measure was on average:
  - Inter-rater:
    - Upper Extremity: 89%
    - Lower Extremity: 91%
  - Intra-rater:
    - Upper Extremity: 81%
    - Lower Extremity: 84%
- **Disagreement** measures: in most cases where values obtained were not within the interval, the gaps are low. Details below and in the following table:
  - **Relative Position** measure met overall the [-0.1, 0.1] interval, being on average:
    - Inter-rater:
      - Upper Extremity: 0.0107
      - Lower Extremity: 0.009
    - Intra-rater:
      - Upper Extremity: 0.03
      - Lower Extremity: 0.036
  - **Relative Concentration** measure met overall the [-0.1, 0.1] interval, being on average:
    - Inter-rater:
      - Upper Extremity: -0.0009
      - Lower Extremity: 0.0224
    - Intra-rater:
      - Upper Extremity: 0.0046
      - Lower Extremity: 0.0035
  - **Relative Variation** measure met overall the [0, 0.1] interval, being on average:
    - Inter-rater:
      - Upper Extremity: 0.0043
      - Lower Extremity: 0.0108
    - Intra-rater:
      - Upper Extremity: 0.0231
      - Lower Extremity: 0.0312

| **Comparison Type** | **Extremity** | **FMA scale part** | **Approach** | **PA** | **RP** | **RC** | **RV** | **Svensson's template used** |
| --- | --- | --- | --- | --- | --- | --- | --- | --- |
| **INTER-rater (KT1 vs KT2)** | **Upper Extremity** | **A** | Day1 | 60% | 0,08 | 0,016536 | 0,012 | VAS scatterplot |
|  |  |  | Day2 | 70% | -0,01 | 0,016162 | 0,012 |  |
|  |  | **B** | Day1 | 80% | 0,04 | -0,13369 | 0 | 11 categories |
|  |  |  | Day2 | 80% | 0,04 | 0,035165 | 0 |  |
|  |  | **C** | Day1 | 90% | 0,05 | 0,065934 | 0,036 | VAS scatterplot |
|  |  |  | Day2 | 100% | 0 | 0 | 0 |  |
|  |  | **D** | Day1 | 90% | -0,03 | 0,09009 | 0 | 11 categories |
|  |  |  | Day2 | 100% | 0 | 0 | 0 |  |
|  |  | **Total A-D (motor function)** | Day1 | 40% | 0,04 | 0,004026 | 0,024 | VAS scatterplot |
|  |  |  | Day2 | 50% | 0,03 | 0,008081 | 0 |  |
|  |  | **H** | Day1 | 100% | 0 |  | 0 | VAS scatterplot |
|  |  |  | Day2 | 100% | 0 |  | 0 |  |
|  |  | **I** | Day1 | 100% | 0 | 0 | 0 | VAS scatterplot |
|  |  |  | Day2 | 90% | -0,01 | -0,06378 | 0 |  |
|  |  | **J** | Day1 | 100% | 0 | 0 | 0 | VAS scatterplot |
|  |  |  | Day2 | 90% | -0,01 | -0,03838 | 0 |  |
|  | **Lower Extremity** | **E** | Day1 | 60% | 0 | 0,064646 | 0,06 | VAS scatterplot |
|  |  |  | Day2 | 70% | -0,01 | 0,012121 | 0,036 |  |
|  |  | **F** | Day1 | 90% | 0,07 | -0,03166 | 0 | 11 categories |
|  |  |  | Day2 | 100% | 0 | 0 | 0 |  |
|  |  | **Total E-F (motor function)** | Day1 | 60% | -0,01 | 0,048701 | 0,168 | VAS scatterplot |
|  |  |  | Day2 | 70% | 0 | -0,06039 | 0,012 |  |
|  |  | **H** | Day1 | 100% | 0 | 0 | 0 | VAS scatterplot |
|  |  |  | Day2 | 100% | 0 | 0 | 0 |  |
|  |  | **I** | Day1 | 100% | 0 | 0 | 0 | VAS scatterplot |
|  |  |  | Day2 | 100% | 0 | 0 | 0 |  |
|  |  | **J** | Day1 | 90% | 0,03 | 0,178571 | 0,012 | VAS scatterplot |
|  |  |  | Day2 | 100% | 0 | 0 | 0 |  |
| **INTRA-rater (Day1 vs Day2)** | **Upper Extremity** | **A** | KT1 | 50% | 0,14 | -0,07083 | 0,192 | VAS scatterplot |
|  |  |  | KT2 | 40% | 0,04 | -0,23133 | 0,012 |  |
|  |  | **B** | KT1 | 80% | -0,04 | -0,03516 | 0 | 11 categories |
|  |  |  | KT2 | 80% | -0,04 | 0,13369 | 0 |  |
|  |  | **C** | KT1 | 80% | 0 | 0,168138 | 0,036 | VAS scatterplot |
|  |  |  | KT2 | 90% | -0,05 | 0,108507 | 0 |  |
|  |  | **D** | KT1 | 70% | 0,09 | 0,101099 | 0,048 | 11 categories |
|  |  |  | KT2 | 80% | 0,12 | 0,008913 | 0,036 |  |
|  |  | **Total A-D (motor function)** | KT1 | 30% | -0,02 | -0,00803 | 0,108 | VAS scatterplot |
|  |  |  | KT2 | 30% | 0,05 | -0,07246 | 0,048 |  |
|  |  | **H** | KT1 | 100% | 0 |  | 0 | VAS scatterplot |
|  |  |  | KT2 | 100% | 0 |  | 0 |  |
|  |  | **I** | KT1 | 90% | 0,01 | 0,063785 | 0 | VAS scatterplot |
|  |  |  | KT2 | 100% | 0 | 0 | 0 |  |
|  |  | **J** | KT1 | 80% | 0,08 | -0,07675 | 0 | VAS scatterplot |
|  |  |  | KT2 | 90% | 0,07 | -0,11513 | 0 |  |
|  | **Lower Extremity** | **E** | KT1 | 50% | 0,1 | -0,08681 | 0,204 | VAS scatterplot |
|  |  |  | KT2 | 50% | 0,1 | -0,18333 | 0,096 |  |
|  |  | **F** | KT1 | 80% | 0,08 | -0,07237 | 0 | 11 categories |
|  |  |  | KT2 | 90% | 0,01 | -0,03906 | 0 |  |
|  |  | **Total E-F (motor function)** | KT1 | 50% | 0,09 | -0,11084 | 0,084 | VAS scatterplot |
|  |  |  | KT2 | 50% | 0,1 | -0,2439 | 0,144 |  |
|  |  | **H** | KT1 | 100% | 0 | 0 | 0 | VAS scatterplot |
|  |  |  | KT2 | 100% | 0 | 0 | 0 |  |
|  |  | **I** | KT1 | 90% | 0,02 | 0,119048 | 0 | VAS scatterplot |
|  |  |  | KT2 | 90% | 0,02 | 0,119048 | 0 |  |
|  |  | **J** | KT1 | 90% | 0,03 | 0,178571 | 0,012 | VAS scatterplot |
|  |  |  | KT2 | 100% | 0 | 0 | 0 |  |

Synthesis Table. Svensson’s method results: inter and intra-rater comparisons
