# Supplementary material for: Translation of the Fugl-Meyer assessment into Romanian: Transcultural and semantic-linguistic adaptations and clinical validation
Source: Front Neurol. 2023 Jan 5;13:1022546. doi: 10.3389/fneur.2022.1022546 (PMC9879050; doi:10.3389/fneur.2022.1022546)
Supplement: Supplementary file 1 [file Data_Sheet_1.ZIP › Suppl.3 - Spearman DataBase and test - with meta-initials.docx]

In order to be able to make the correlation test, afferent to objectifing concurrent validity FMA score values, these were computed by averaging the score values obtained 4 times (2 times by each KT- see the Materials and Methods section in the body text ) regarding total Upper Extremity A-D (motor function), and similarly, the Lower Extremity total E-F (motor function). The correlation values obtained were very good with BI, and at limits for the mRS (with p-values close to the 0,05 statistical significance threshold, considering that the number of records was only 10, and the small number of items i.e. 7, of the mRS). Note: *rho* stands for the Spearman's rank correlation rho coefficient.

|  | **FMA UE A-D** | | **FMA LE E-F** | |
| --- | --- | --- | --- | --- |
|  | rho | p-value | rho | p-value |
| **BI** | 0,916962 | 0,000188 | 0,631949 | 0,04998 |
| **mRS** | -0,59367 | 0,07039 | -0,6615 | 0,03724 |

Synthesis tabel Spearman correletion test results.

Input data for the correlation of the two FMA total motor function scores values (Upper Extremity A-D and Lower Extremity E-F) against the BI and the mRS, via Spearman correlation (Synthesis table primary data):

| **Crt. no.** | **Meta Initials** | **Initials** | **BI** | **mRS** | **FMA_UE A-D_average** | **FMA_LE E-F_average** |
| --- | --- | --- | --- | --- | --- | --- |
| 1 | PO | MV | 40 | 3 | 44 | 27,25 |
| 2 | VD | TM | 40 | 3 | 44 | 25 |
| 3 | TA | SM1 | 30 | 4 | 32,25 | 11,5 |
| 4 | HB | TE | 35 | 4 | 21,5 | 27,5 |
| 5 | RD | SM2 | 48 | 3 | 56 | 29 |
| 6 | CN | SM3 | 49 | 3 | 62 | 30 |
| 7 | AF | ID | 40 | 4 | 61,25 | 16,5 |
| 8 | BL | TD | 20 | 4 | 0 | 17,25 |
| 9 | DM | MG | 39 | 3 | 40 | 26,5 |
| 10 | SC | AM | 38 | 4 | 14 | 23 |

Correlation plots for FMA against BI and against mRS:
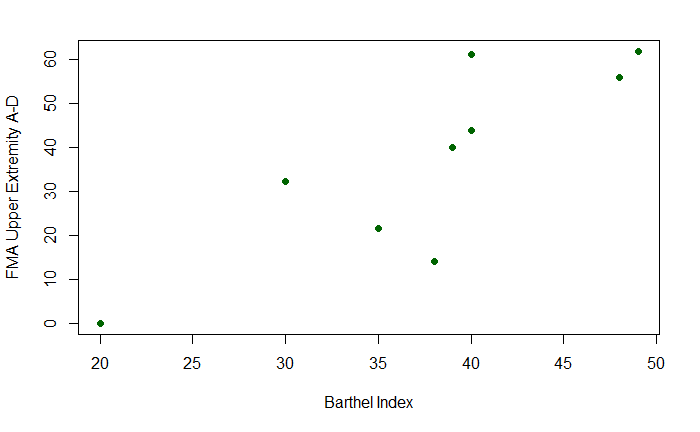


Figure 1


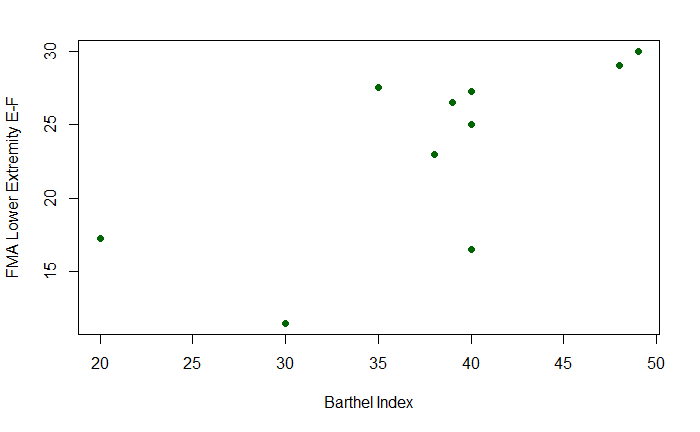


Figure 2


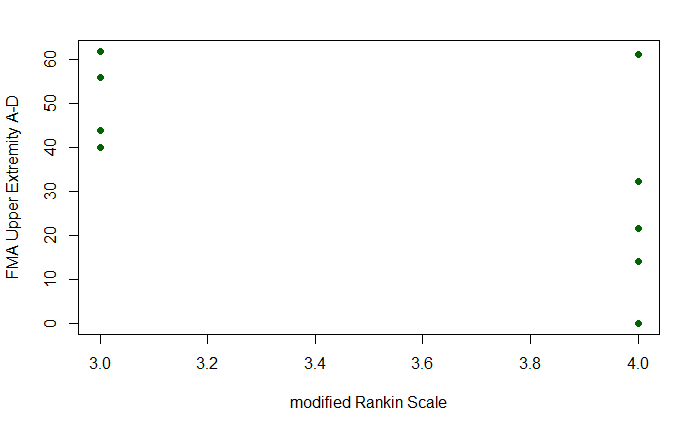


Figure 3


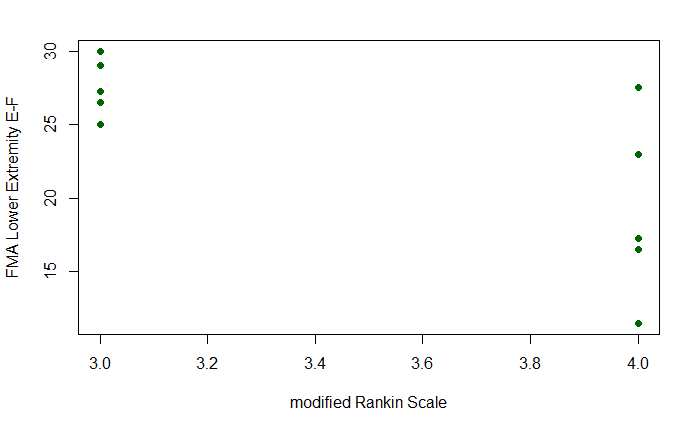


Figure 4
