# Supplementary material for: Translation of the Fugl-Meyer assessment into Romanian: Transcultural and semantic-linguistic adaptations and clinical validation
Source: Front Neurol. 2023 Jan 5;13:1022546. doi: 10.3389/fneur.2022.1022546 (PMC9879050; doi:10.3389/fneur.2022.1022546)
Supplement: Supplementary file 1 [file Data_Sheet_1.ZIP › Annex 1 1723674_fm-le-eng-190303-protocol.pdf]

FUGL-MEYER ASSESSMENT LOWER EXTREMITY (FMA-LE) Assessment of sensorimotor function

ID:
Date:
Examiner:

Fugl-Meyer AR, Jaasko L, Leyman I, Olsson S, Steglind S: The post-stroke hemiplegic patient. 1. a method for evaluation of physical performance. Scand J Rehabil Med 1975, 7:13-31.

E. LOWER EXTREMITY

**I. Reflex activity**, supine position		none	can be elicited
**Flexors**: knee flexors		0	2
**Extensors**: patellar, achilles (at least one)		0	2
	Subtotal I (max 4)		

**II. Volitional movement within synergies** supine position			none	partial	full
**Flexor synergy:** Maximal hip flexion (abduction/external rotation), maximal flexion in knee and ankle joint (palpate distal tendons to ensure active knee flexion).	Hip	flexion	0	1	2
	Knee	flexion	0	1	2
	Ankle	dorsiflexion	0	1	2
**Extensor synergy:** From flexor synergy to the hip extension/adduction, knee extension and ankle plantar flexion. Resistance is applied to ensure active movement, evaluate both movement and strength (compare with the unaffected side)	Hip	extension	0	1	2
		adduction	0	1	2
	Knee	extension	0	1	2
	Ankle	plantar flexion	0	1	2
		Subtotal II (max 14)			

**III. Volitional movement mixing synergies** sitting position, knee 10cm from the edge of the chair/bed		none	partial	full
**Knee flexion** from actively or passively extended knee	no active motion<br>less than 90° active flexion, palpate tendons of hamstrings<br>more than 90° active flexion	0	1	2
**Ankle dorsiflexion** compare with unaffected side	no active motion<br>limited dorsiflexion<br>complete dorsiflexion	0	1	2
	Subtotal III (max 4)			

**IV. Volitional movement with little or no synergy** standing position, hip at 0°		none	partial	full
**Knee flexion to 90°** hip at 0°, balance support is allowed	no active motion or immediate, simultaneous hip flexion<br>less than 90° knee flexion and/or hip flexion during movement<br>at least 90° knee flexion without simultaneous hip flexion	0	1	2
**Ankle dorsiflexion** compare with unaffected side	no active motion<br>limited dorsiflexion<br>complete dorsiflexion	0	1	2
	Subtotal IV (max 4)			

**V. Normal reflex activity** supine position, assessed only if full score of 4 points is achieved in part IV, compare with the unaffected side		hyper	lively	normal
**Reflex activity** knee flexors, Patellar, Achilles,	2 of 3 reflexes markedly hyperactive<br>1 reflex markedly hyperactive or at least 2 reflexes lively<br>maximum of 1 reflex lively, none hyperactive	0	1	2
	Subtotal V (max 2)			
	**Total E** (max 28)			

GÖTEBORGS UNIVERSITET

**F. COORDINATION/SPEED**, supine, after one trial with both legs, eyes closed, heel to knee cap of the opposite leg, 5 times as fast as possible		**marked**	**slight**	**none**
**Tremor**	at least 1 completed movement	0	1	2
**Dysmetria**	pronounced or unsystematic<br>slight and systematic<br>no dysmetria	0	1	2
		**≥ 6s**	**2 - 5s**	**< 2s**
**Time**<br>start and end with the hand on the knee	6 or more seconds slower than unaffected side<br>2-5 seconds slower than unaffected side<br>less than 2 seconds difference	0	1	2
	**Total F** (max 6)			

**H. SENSATION**, lower extremity<br>eyes closed, compare with the unaffected side		**anesthesia**	**hypoesthesia or dysesthesia**	**normal**
**Light touch**	leg<br>foot sole	0<br>0	1<br>1	2<br>2
		**less than 3/4 correct or absence**	**3/4 correct or considerable difference**	**correct 100%, little or no difference**
**Position**<br>small alterations in the position	hip<br>knee<br>ankle<br>great toe (IP-joint)	0<br>0<br>0<br>0	1<br>1<br>1<br>1	2<br>2<br>2<br>2
			**Total H** (max12)	

**I. PASSIVE JOINT MOTION**, lower extremity<br>supine position, compare with the unaffected side					**J. JOINT PAIN** during passive motion, lower extremity		
		only few degrees (<10° hip)	decreased	normal	pronounced pain during movement or very marked pain at the end of the movement	some pain	no pain
**Hip**	Flexion<br>Abduction<br>External rotation<br>Internal rotation	0<br>0<br>0<br>0	1<br>1<br>1<br>1	2<br>2<br>2<br>2	0<br>0<br>0<br>0	1<br>1<br>1<br>1	2<br>2<br>2<br>2
**Knee**	Flexion<br>Extension	0<br>0	1<br>1	2<br>2	0<br>0	1<br>1	2<br>2
**Ankle**	Dorsiflexion<br>Plantar flexion	0<br>0	1<br>1	2<br>2	0<br>0	1<br>1	2<br>2
**Foot**	Pronation<br>Supination	0<br>0	1<br>1	2<br>2	0<br>0	1<br>1	2<br>2
**Total** (max 20)					**Total** (max 20)		

**E. LOWER EXTERMTY**	/28
**F. COORDINATION / SPEED**	/6
**TOTAL E-F (motor function)**	/34

**H. SENSATION**	/12
**I. PASSIVE JOINT MOTION**	/20
**J. JOINT PAIN**	/20

GÖTEBORGS UNIVERSITET
