# Supplementary material for: Translation of the Fugl-Meyer assessment into Romanian: Transcultural and semantic-linguistic adaptations and clinical validation
Source: Front Neurol. 2023 Jan 5;13:1022546. doi: 10.3389/fneur.2022.1022546 (PMC9879050; doi:10.3389/fneur.2022.1022546)
Supplement: Supplementary file 1 [file Data_Sheet_1.ZIP › ANNEX 2 - Official in English, translated into Romanian, FMA scale’s evaluation protocols.docx]

PROTOCOL de EVALUARE FUGL-MEYER (EFM) ^Medicină de Recuperare, Universitatea din Göteborg^

^(Gothenburg University)^

**EVALUAREA FUGL**-**MEYER Identitate Pacient**:

**EXTREMITATEA SUPERIOARĂ** (**EFM**-**ES**) **Data**:

**Evaluarea funcției senzitivomotorii Examinator**:

*Fugl-Meyer AR, Jaasko L, Leyman I, Olsson S, Steglind S: The post-stroke hemiplegic patient. A method for evaluation of physical performance. Scand J Rehabil Med 1975, 7:13-31.*

| **A. EXTREMITATEA SUPERIOARĂ,** din poziție șezând | | | | | | | | | | | | |  |
| --- | --- | --- | --- | --- | --- | --- | --- | --- | --- | --- | --- | --- | --- |
| **I. Activitate reflexă** | | | | **absentă** | | | | | **poate fi provocată** | | | |  |
| **Flexori**: biceps și flexori degete (cel puțin unul)  **Extensori**: triceps | | | | 0  0 | | | | | 2  2 | | | |  |
| Subtotal I (max 4) | | | |  | | | | | | | | |  |
| **II. Mișcare voluntară în cadrul sinergiilor**, fară ajutor gravitațional | | | | **absentă** | | **parțială** | | | | | **completă** | |  |
| **Sinergia de flexie**: Mâna (dusă) de la genunchiul contralateral către urechea ipsilaterală. Din sinergia de extensie (adductie umăr/rotație internă, extensie cot, pronație antebraț) spre sinergia de flexie (abducție umăr/rotație externă, flexie cot, supinație antebraț) | | | Umăr retropulsie  ridicare  abducție 90^0^  rotație externă^1^  Cot flexie Antebrat supinație | 0  0  0  0  0  0 | | 1  1  1  1  1  1 | | | | | 2  2  2  2  2  2 | |  |
| **Sinergia de extensie**: Mâna (dusă) de la urechea ipsilaterală spre genunchiul contralateral^2^ | | | Umăr adducție/rotație internă^2^  Cot extensie  Antebraț pronație | 0  0  0 | | 1  1  1 | | | | | 2  2  2 | |  |
| Subtotal II (max 18) | | | |  | | | | | | | | |  |
| **III. Mișcare voluntară combinând sinergiile**, fără compensare | | | | **absentă** | | **parțială** | | | | **completă** | | |  |
| **Mâna** (dusă) **către coloana lombară**;  (se incepe cu) mana aflată în poală | nu poate efectuă/mâna în fața SIAS  mâna în spatele SIAS (fără compensare)  mâna (dusă) la nivelul coloanei (fără compensare) | | | 0 | | 1 | | | | 2 | | |  |
| **Flexie umăr** (articulația scapulo-humerală) **0**-**90**°  cot la 0°  pronație-supinație 0° | abducție imediată (la inițierea mișcării) sau flexie cot  abductie (brat) sau flexie cot în cursul mișcării  flexie 90°, fără abducție umăr sau flexie cot | | | 0 | | 1 | | | | 2 | | |  |
| **Pronație**-**supinație**  cot la 90^0^  umăr la 0^0^ | fără pronație/supinație, poziție de start imposibilă (nu poate fi menținută)  pronatia/supinația limitată, menține poziția de start,  pronație/supinație completă, menține poziția de start | | | 0 | | 1 | | | | 2 | | |  |
| Subtotal III (max 6) | | | |  | | | | | | | | |  |
|  | | | |  | | | | | | | | |  |
| **IV. Mișcare voluntară cu sinergie scazută sau absenta** (**inaparentă**) | | | | **absentă** | | **parțială** | | | | **completă** | | |  |
| **Abducție umăr** (articulația scapulo-humerală) **0**-**90°**  cot la 0°  antebraț neutru | | supinație sau flexie cot imediată (la inițierea mișcării)  supinație sau flexie cot în timpul mișcării  abducție 90°, menține extensie cot și pronație antebraț | | | 0 | | 1 | | | | 2 | | |
| **Flexie umăr** (articulația scapulo-humerală) **90**-**180°**  cotul la 0°  pronație-supinație 0^0^ | | abducție sau flexie cot imediată (la inițierea mișcării)  abducție (braț) sau flexie cot în cursul mișcării  flexie 180^0^, fără abducție umăr sau flexie cot^3^ | | | 0 | | 1 | | | | 2 | | |
| **Pronație**/ **supinatie**  cot la 0°  umăr în flexie la 30-90° | | fără pronație/supinație, poziție de start imposibilă (nu poate fi menținută)^4^  pronație/supinație limitate, menține poziția de start  pronație/supinație completă, menține poziția de start | | | 0 | | 1 | | | | 2 | | |
| Subtotal IV (max 6) | | | |  | | | | | | | | |  |
| **V. Activitate reflexă normală**, se evaluează numai dacă scorul obținut în partea (pasul) IV este de 6 puncte; comparați cu partea neafectată | | | | **hiperactiv** | | | | **viu** | | | | **normal** |  |
| Bicipital, tricipital, al flexorilor degetelor^5^ | | 2 din 3 reflexe marcat hiperactive  1 reflex marcat hiperactiv sau cel puțin 2 reflexe vii  maximum 1 reflex viu, nici unul hiperactiv | | 0 | | | | 1 | | | | 2 |  |
| Subtotal V (max 2) | | | |  | | | | | | | | |  |
| **Total A** (max 36) | | | |  | | | | | | | | |  |

| **B. Încheietura mâinii** (articulația pumnului/radio-carpiană), poate fi susținut cotul (1/3 proximală antebraț) pentru a lua/a menține poziția de start, fără sprijin la nivelul încheieturii mâinii (articulația pumnului/radio-carpiană), verificati amplitudinea miscarii pasive înainte de testare | | **absentă** | **parțială** | **completă** |
| --- | --- | --- | --- | --- |
| **Stabilitate la dosiflexie 15^0^**  cot la 90^0^, antebraț pronat  umăr la 0^0^ | mai puțin de 15° dorsiflexie activă  dorsiflexie 15°, nu tolerează/nu poate efectua contra unei rezistențe  menține dorsiflexia împotriva unei rezistențe | 0 | 1 | 2 |
| **Dorsiflexie**/**flexie volară repetată**  cot la 90^0^  antebraț pronat  umăr la 0^0^,  ușoară flexie a degetelor | nu se poate efectua voluntar  amplitudine de mișcare activă limitată  amplitudine de mișcare activă completă (efectuata lin/bine) | 0 | 1 | 2 |
| **Stabilitate la dorsiflexie 15^0^**  cot la 0^0^, antebraț pronat  ușoară flexie/abducție umăr | mai putin de 15° dorsiflexie activă  dorsiflexie 15°, nu tolerează/nu poate efectua contra unei rezistențe  menține dorsiflexia împotriva unei rezistențe | 0 | 1 | 2 |
| **Dorsiflexie**/**flexie volară repetată**  cot la 0^0^,  antebraț pronat  ușoară flexie/abducție umăr | nu se poate efectua voluntar  amplitudine de mișcare activă limitată  amplitudine de mișcare activă completă (efectuata lin/ bine) | 0 | 1 | 2 |
| **Circumducție**  cot la 90^0^,  antebraț pronat  umăr la 0^0^ | nu se poate efectua voluntar  miscare nearmonioasa sau incompleta  circumductie completa, efectuata lin/bine | 0 | 1 | 2 |
| **Total B** (max 10) | |  | | |

| **C. MÂNA**, poate fi susținut cotul (1/3 proximală antebrat) pentru a mentine 90^0^ flexie, fără sprijin la nivelul încheieturii mâinii (articulația pumnului/radio-carpiană), comparați cu mana neafectata , se interpun obiecte pentru prehensiune/prindere activă | | **absentă** | **parțială** | **completă** |
| --- | --- | --- | --- | --- |
| **Flexie globală**/**în bloc** (a tuturor degetelor) din poziție de extensie completă activă sau pasivă (antebraț în poziție neutră) |  | 0 | 1 | 2 |
| **Extensie globală**/**în bloc (**a tuturor degetelor) din poziție de flexie completă activă sau pasivă (antebraț în poziție neutră) |  | 0 | 1 | 2 |
| **PREHENSIUNE** | | | | |
| **a. Prehensiune**/**prindere cârlig**  flexie în articulațiile interfalangiene proximale și distale (degetele II-V), extensie în articulațiile metacarpofalangiene II-V (antebraț in poziție neutră) | nu se poate efectua voluntar  se mentine pozitia impotriva unei rezistente, dar slab  se mentine pozitia impotriva unei rezistente | 0 | 1 | 2 |
| **b. Adducție police**  prima articulație caropometacarpiană, metacarpofalangiană, interfalangiană la 0^0^, bucată de hârtie (introdusă) între police și a doua articulație metacarpofalangiană (antebraț în poziție semi-pronată) | nu se poate efectua voluntar  poate tine hartia, dar nu impotriva tragerii acesteia  poate tine hartia si impotriva tragerii acesteia | 0 | 1 | 2 |
| **c. Pensa** bidigitală, **opoziție** pulpa policelui față de pulpa degetului 2, se trage în sus un creion (ținut de pacient între degetele respective) (antebraț în poziție neutră) | nu se poate efectua  poate ține creionul, dar nu impotriva tragerii acestuia  poate ține creionul și împotriva tragerii acestuia | 0 | 1 | 2 |
| **d. Priza cilindrică**  obiect de formă cilindrică (cană mică) trasă în sus (dintre police și degete – aflat în opoziție cu degetele – respectiv polidigito-palmară; antebraț in poziție neutră) | nu se poate efectua  poate tine cilindrul dar nu impotriva smulgerii acesteia  poate tine cilindrul si impotriva smulgerii acestuia | 0 | 1 | 2 |
| **e. Priza sferică**  degetele in abducție/flexie, policele in opoziție, minge de tenis, trasă în afară (antebraț in poziție neutră) | nu se poate efectua  poate ține sfera dar nu impotriva tragerii acesteia  poate ține sfera și impotriva tragerii acesteia | 0 | 1 | 2 |
| **Total C** (max 14) | |  | | |

| **D.** (**DIS**)**COORDONARE**/**VITEZĂ**, din pozțtie șezând, după o testare la ambele membre superioare, ochii închiși, vârful indexului (deplasat) de la genunchiul ipsilateral la nas de 5 ori, cât mai rapid posibil | | **marcat** | **ușor** | **absent** |
| --- | --- | --- | --- | --- |
| **Tremor** |  | 0 | 1 | 2 |
| **Dismetrie** | pronunțată sau nesistematică (nesistematizată)  ușoară și sistematică (sistematizată)  fără dismetrie | 0 | 1 | 2 |
|  |  | **≥ 6s** | **2**-**5s** | **< 2s** |
| **Timp**  începere și terminare cu mâna pe genunchi | cu 6 secunde sau mai mult, mai lent decat pe partea neafectată  cu 2-5 secunde mai lent decat pe partea neafectată  mai puțin de 2 secunde diferență | 0 | 1 | 2 |
| **Total D** (max 6) | |  | | |

| **TOTAL A-D** (max 66) | | | |  | |
| --- | --- | --- | --- | --- | --- |
| **H. SENSIBILITATE**, extremitatea superioară, ochii închiși, comparați cu partea neafectată | | **anestezie** | **hipoestezie sau disestezie** | | **normal** |
| **Atingere ușoară** | braț, antebraț  fața palmară a mâinii | 0  0 | 1  1 | | 2  2 |
|  |  | **mai puțin de 3**/**4 sau absența** (**sensibilității**) | **3**/**4 corectă**  **sau diferență**  **considerabiă** | | **corectă100%**, **diferență mică sau fără diferență** |
| **Poziție**  alterări ușoare ale poziției | umăr  cot  încheietura mâinii (articulația pumnului/radio-carpiană)  police (articulația interfalangiană) | 0  0  0  0 | 1  1  1  1 | | 2  2  2  2 |
| **Total H** (max12) | | | | |  |

| **I. MOBILITATE ARTICULARĂ PASIVĂ**, extremitatea superi-oară, (din poziție șezând), comparați cu partea neafectată | | | | **J. DURERE ARTICULARĂ,** în timpul mișcării pasive, extremitatea superioară | | |
| --- | --- | --- | --- | --- | --- | --- |
|  | doar câteva grade  (mai puțin de 10° în umăr) | scazută (între 10° – spre exp. în umăr – și val. norm. în artic. resp.) | normală | durere pronunțată în timpul mișcării sau foarte marcată la finalul mișcării | puțină/  ușoară durere | fară durere |
| **Umăr** (articulația scapulo-humerală)  Flexie (0° - 180°)  Abducție (0°-90°)  Rotație externă  Rotație internă | 0  0  0  0 | 1  1  1  1 | 2  2  2  2 | 0  0  0  0 | 1  1  1  1 | 2  2  2  2 |
| **Cot**  Flexie  Extensie | 0  0 | 1  1 | 2  2 | 0  0 | 1  1 | 2  2 |
| **Antebraț**  Pronație  Supinație | 0  0 | 1  1 | 2  2 | 0  0 | 1  1 | 2  2 |
| Încheietura mâinii (articulația pumnului/ radio-carpiană)  Flexie  Extensie | 0  0 | 1  1 | 2  2 | 0  0 | 1  1 | 2  2 |
| **Degete**  Flexie  Extensie | 0  0 | 1  1 | 2  2 | 0  0 | 1  1 | 2  2 |
| **Total** (max 24) | | | | **Total** (max 24) | | |

| **A. EXTREMITATEA SUPERIOARĂ** | /36 |
| --- | --- |
| **B.** Încheietura mâinii (articulația pumnului/radio-carpiană) | /10 |
| **C. MÂNA** | /14 |
| **D.** (**DIS**)**COORDONARE**/**VITEZĂ** | /6 |
| **TOTAL A**-**D** (**funcție motorie**) | /66 |

| **H. SENSIBILITATE** | /12 |
| --- | --- |
| **I. MOBILITATE ARTICULARĂ PASIVĂ** | /24 |
| **J. DURERE ARTICULARĂ** | /24 |

(Afferent to the FMA-Upper Extremity – UE), i. e.: Aferent la evaluarea Fugl-Meyer – extremitatea superioară:

1. abducție și rotatie externă – articulața scapulo-humerală

2. poate fi susținut brațul pentru a lua/menține poziția de start

- prezența doar a mișcărilor compensatorii în locul celor active se cuantifică/punctează cu

0 (exemple:

3. flexie antebraț și pronație, la flexia brațului/umăr 90-180°;

4. abducție și flexie cot la supinație)

5. mâna în pronație, degetele pacientului in semiflexie MCF și IF, sprijinite relaxat pe indexul și mediusul examinatorului, cand acesta își percută de jos în sus cu ciocanul de reflexe propriile degete, se poate obține o flexie a degetelor pacientului, aspect posibil normal

PROTOCOL de EVALUARE FUGL-MEYER (EFM) ^Medicină de Recuperare, Universitatea din Göteborg^

^(Gothenburg University)^

**EVALUAREA FUGL**-**MEYER Identitate Pacient**:

**EXTREMITATEA INFERIOARĂ** (**EFM**-**EI**) **Data**:

**Evaluarea funcției senzitivomotorii Examinator**:

*Fugl-Meyer AR, Jaasko L, Leyman I, Olsson S, Steglind S: The post-stroke hemiplegic patient. A method for evaluation of physical performance. Scand J Rehabil Med 1975, 7:13-31.*

| **E. EXTREMITATEA INFERIOARĂ** | | | | | | | | | | | |
| --- | --- | --- | --- | --- | --- | --- | --- | --- | --- | --- | --- |
| **I. Activitate reflexă**, din decubit dorsal | | | | | | **absentă** | | **poate fi provocată** | | | |
| **Flexori**: flexorii genunchiului (reflex ischiogambier - „hamstrings”)  **Extensori**: patelar, achilian (cel puțin unul) | | | | | | 0  0 | | 2  2 | | | |
| Subtotal I (max 4) | | | | | |  | | | | | |
| **II. Mișcare voluntară în cadrul sinergiilor,** din decubit dorsal | | | | | | **absentă** | **parțială** | | | **completă** | |
| **Sinergia flexorilor:** Flexie maximă șold (abducție/rotație externă), flexie maximă în articulațiile genunchiului și gleznei (se palpează distal tendoanele pentru a se asigura de flexia activă a genunchiului). | | | | Șold  Genunchi  Gleznă | flexie^1^  flexie  dorsiflexie | 0  0  0 | 1  1  1 | | | 2  2  2 | |
| **Sinergia extensorilor**: De la sinergia de flexie până la extensie șold, extensie genunchi și flexie plantară gleznă. Se aplică ușoară rezistență la nivelul extremității distale fața postero-medială coapsă, pentru a ne asigura de mișcarea activă, evaluați atât mișcarea cât și forța (comparați cu partea neafectată) | | | | Șold  Genunchi  Glezna | extensie  adductie  extensie  flexie plantara | 0  0  0  0 | 1  1  1  1 | | | 2  2  2  2 | |
| Subtotal II (max 14) | | | | | |  | | | | | |
| **III. Mișcare voluntară combinând sinergiile**, din poziție șezând, genunchi la 10 cm distal de marginea patului/scaunului | | | | | | **absentă** | **parțială** | | | **completă** | |
| **Flexie genunchi** de la genunchi extins activ sau pasiv^2^ | fără mișcare activă  flexie activă mai puțin de 90°, se palpează tendoanele ischiogambierilor („hamstrings”)  flexie activă mai mult de 90° | | | | | 0 | 1 | | | 2 | |
| **Dorsiflexie gleznă** comparați cu partea neafectată | fără mișcare activă  dorsiflexie limitată  dorsiflexie completă | | | | | 0 | 1 | | | 2 | |
| Subtotal III (max 4) | | | | | |  | | | | | |
| **IV. Miscare voluntară cu sinergie scazută sau absentă** (**inaparentă**), din ortostatism, șold la 0^0^ | | | | | | **absentă** | **parțială** | | | **completă** | |
| **Flexie genunchi la 90**^0^  șold la 0^0^, sunt permise asistența/sprijinul pentru echilibru | | fără mișcare activă sau flexie imediată (la inițierea mișcării), simultană șold  mai putin de 90° flexie genunchi sau/și flexie șold în timpul mișcării  flexie genunchi cel putin 90°, fără flexie simultană a șoldului | | | | 0 | 1 | | | 2 | |
| **Dorsiflexie gleznă**  comparați cu partea neafectată | | fără mișcare activă  dorsiflexie limitată  dorsiflexie completă | | | | 0 | 1 | | | 2 | |
| Subtotal IV (max 4) | | | | | |  | | | | | |
| **V. Activitate reflexă normală**, din decubit dorsal, evaluați numai dac scorul obținut în partea (pasul) IV este de 4 puncte; comparați cu partea neafectată | | | | | | **hiperactiv** | | | **viu** | | **normal** |
| **Activitate reflexă** flexorii genunchiului (reflex ischiogambier -„hamstrings”), patelar, ahilian | | | 2 din 3 reflexe marcat hiperactive  1 reflex marcat hiperactiv sau cel puțin 2 reflexe vii  maximum un reflex viu, nici unul hiperactiv | | | 0 | | | 1 | | 2 |
| Subtotal V (max 2) | | | | | |  | | | | | |
| **Total E** (max 28) | | | | | |  | | | | | |

| **F.** (**DIS**)**COORDONARE**/**VITEZĂ,** din decubit dorsal, după o testare cu ambele membre inferioare, ochii inchiși, călcâiul la rotula membrului inferior de parte opusă, de 5 ori cât mai rapid posibil | | **marcat** | **ușor** | **absent** |
| --- | --- | --- | --- | --- |
| **Tremor** |  | 0 | 1 | 2 |
| **Dismetrie** | pronunțată sau nesistematică (nesistematizată)  ușoară și sistematică (sistematizată)  fără dismetrie | 0 | 1 | 2 |
|  |  | **≥ 6s** | **2**-**5s** | **< 2s** |
| **Timp** | cu 6 secunde sau mai mult, mai lent față de partea neafectată  cu 2-5 secunde mai lent față de partea neafectată  mai puțin de 2 secunde diferență | 0 | 1 | 2 |
| **Total F** (max 6) | |  | | |

| **H. SENSIBILITATE**, extremitatea inferioară, ochii închiși, comparați cu partea neafectată | | **anestezie** | **hipoestezie sau disestezie** | **normal** |
| --- | --- | --- | --- | --- |
| **Atingere ușoară** | fața dorsală picior  plantă | 0  0 | 1  1 | 2  2 |
|  |  | **mai puțin de 3**/**4 corectă sau absentă** (sensibilitatea) | **3**/**4 corectă sau diferență considerabilă** | **100% corectă**, **diferență mică sau fără diferență** |
| **Poziție** (deposturare)  alterări ușoare ale poziției | șold  genunchi  gleznă  haluce (articulația interfalangiană) | 0  0  0  0 | 1  1  1  1 | 2  2  2  2 |
| **Total H** (max12) | | | |  |

| **I. MOBILITATE ARTICULARĂ PASIVĂ**, extremitatea inferioara, decubit dorsal, comparați cu partea neafectată | | | | | **J. DURERE ARTICULARĂ** în timpul mișcării pasive, extremitatea inferioară | | |
| --- | --- | --- | --- | --- | --- | --- | --- |
|  | | doar câteva grade (<10° la nivelul șoldului, cvasiimposibilă) | scăzută  (între 10° – spre exp. în șold – și val. norm. în artic. resp.) | normală | durere pronuntață în timpul mișcării sau foarte marcată la finalul mișcării | puțină /ușoară durere | fără durere |
| **Șold** | Flexie  Abducție  Rotație externă Rotație internă | 0  0  0  0 | 1  1  1  1 | 2  2  2  2 | 0  0  0  0 | 1  1  1  1 | 2  2  2  2 |
| **Genunchi** | Flexie  Extensie | 0  0 | 1  1 | 2  2 | 0  0 | 1  1 | 2  2 |
| **Gleznă** | Dorsiflexie  Flexie plantară | 0  0 | 1  1 | 2  2 | 0  0 | 1  1 | 2  2 |
| **Picior** | Pronație  Supinație | 0  0 | 1  1 | 2  2 | 0  0 | 1  1 | 2  2 |
| **Total** (max 20) | | | | | **Total** (max 20) | | |

| **E. EXTREMITATE INTERIOARĂ** | /28 |
| --- | --- |
| **F.** (**DIS**)**COORDONARE**/**VITEZĂ** | /6 |
| **TOTAL E**-**F** (funcție motorie) | /34 |
|  | |
| **H. SENSIBILITATE** | /12 |
| **I. MOBILITATE ARTICULARĂ PASIVĂ** | /20 |
| **J. DURERE ARTICULARĂ** | /20 |

(Afferent to the FMA-Lower Extremity – LE), i. e.: Aferent la evaluarea Fugl-Meyer – extremitatea inferioară:

1. abducție/rotație externă considerate a fi parțiale – prezența doar a respectivelor mișcări

compensatorii în locul celor active se cuantifică / punteaza cu 0

2. se aplică ușoară rezistență distal posterior, la nivelul călcâiului, pentru a ne asigura de

mișcare activă
